# Biosynthesis of Tetrapyrrole Cofactors by Bacterial Community Inhabiting Porphyrine-Containing Shale Rock (Fore-Sudetic Monocline)

**DOI:** 10.3390/molecules26216746

**Published:** 2021-11-08

**Authors:** Robert Stasiuk, Tomasz Krucoń, Renata Matlakowska

**Affiliations:** 1 Department of Geomicrobiology, Institute of Microbiology, Faculty of Biology, University of Warsaw, 02-096 Warsaw, Poland; r.matlakowska@uw.edu.pl; 2 Department of Environmental Microbiology and Biotechnology, Institute of Microbiology, Faculty of Biology, University of Warsaw, 02-096 Warsaw, Poland; tkrucon@biol.uw.edu.pl

**Keywords:** porphyrin, heme, siroheme, cobalamin, shale rock, bacteria, *Pseudomonas*

## Abstract

This study describes for the first time the comprehensive characterization of tetrapyrrole cofactor biosynthetic pathways developed for bacterial community (BC) inhabiting shale rock. Based on the genomic and proteomic metadata, we have detailed the biosynthesis of siroheme, heme, cobalamin, and the major precursor uroporphyrinogen III by a deep BC living on a rock containing sedimentary tetrapyrrole compounds. The obtained results showed the presence of incomplete heme and cobalamin biosynthesis pathways in the studied BC. At the same time, the production of proteins containing these cofactors, such as cytochromes, catalases and sulfite reductase, was observed. The results obtained are crucial for understanding the ecology of bacteria inhabiting shale rock, as well as their metabolism and potential impact on the biogeochemistry of these rocks. Based on the findings, we hypothesize that the bacteria may use primary or modified sedimentary porphyrins and their degradation products as precursors for synthesizing tetrapyrrole cofactors. Experimental testing of this hypothesis is of course necessary, but its evidence would point to an important and unique phenomenon of the tetrapyrrole ring cycle on Earth involving bacteria.

## 1. Introduction

Many cofactors of key bacterial enzymes are derived from tetrapyrrole and contain a core, complexed metal ion (Fe^2+^, Co^2+^, Mg^2+^, and Ni^2+^). A well-known example is iron-containing heme (C_34_H_32_FeN_4_O_4_), which is a prosthetic group in cytochromes, catalases, and peroxidases. Another example is heme-like iron-containing siroheme (C_42_H_36_FeN_4_O_16_). It is a cofactor at the active site of sulfite reductase, a key enzyme in sulfur assimilation pathway that converts sulfite to sulfide, which is then integrated into the organic compound homocysteine [1]. Another important tetrapyrrole cofactor is cobalt-containing cobalamin (vitamin B12) (C_63_H_88_CoN_14_O_14_P). Cobalamin serves as a coenzyme in the synthesis of nucleotides and amino acids, degradation of organic compounds (e.g., alcohols and amines), dehalogenation, fermentation, and regulation of gene expression [2,3]. Cobalamin-dependent enzymes include, but are not limited to, ribonucleotide reductase, methyltransferases, and reductive dehalogenases [4,5,6]. Nickel-containing coenzyme F_430_ (C_42_H_51_N_6_NiO_13_) is a cofactor of methyl coenzyme M reductase, an enzyme involved in the formation of methane [7].

**Figure 1:**
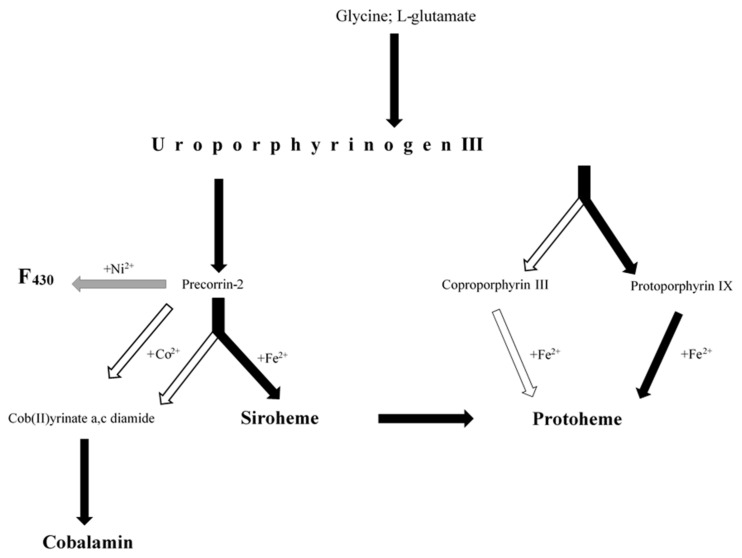
Biosynthetic pathways of tetrapyrrole cofactors (protoheme, siroheme, cobalamin and F_430_) investigated in this study, including the most important precursors (glycine; L-glutamate, uroporphyrinogen III) and intermediates (coproporphyrin III, protoporphyrin IX, precorrin-2, cob(II)yrinate a,c diamide). A detailed description of the pathways is included in the manuscript and in Supplementary Materials (Table S1 and Figures S1–S8). The colour of the arrows indicates whether the pathways have been fully detected (black), partially detected (white) or undetected (grey).

Microorganisms synthesize tetrapyrroles either by de novo synthesis or by scavenging them from the environment. The biosynthesis of tetrapyrrole is one of the fundamental pathways in living organisms, and the biosynthetic pathways of the abovementioned four porphyrin cofactors are closely related (Figure 1). Uroporphyrinogen III (C_40_H_44_N_4_O_16_) is a common precursor of tetrapyrrole cofactors, which is converted to precorrin-2 (C_42_H_48_N_4_O_16_) or protoporphyrin IX (C_34_H_34_N_4_O_4_) (PPIX). Precorrin-2 in turn acts as a precursor of siroheme and vitamin B12 (while PPIX acts as a direct precursor of heme (protoporphyrin-dependent pathway). Heme can also be synthesized from coproporphyrin (coproporphyrin-dependent path way) and siroheme (siroheme-dependent pathway).

**Figure 2:**
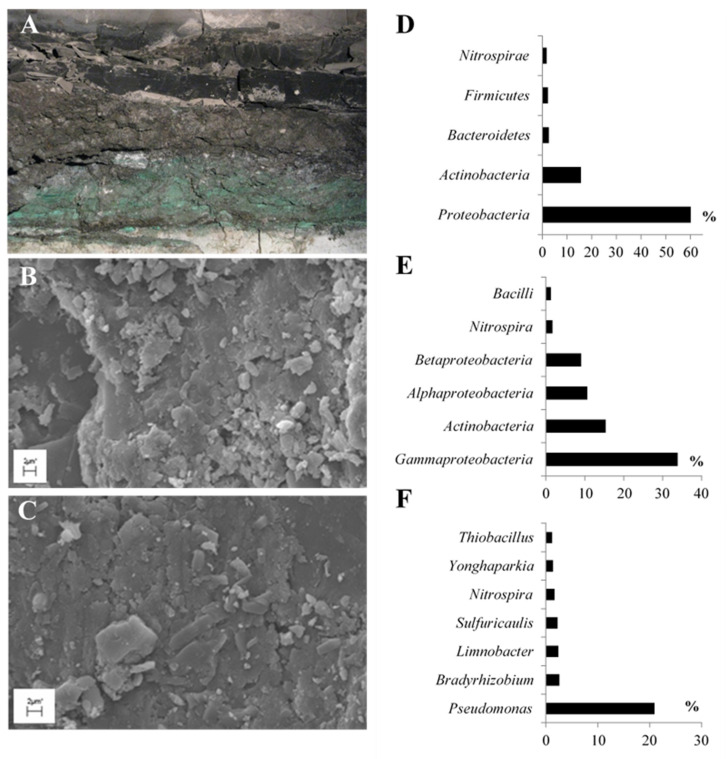
Bacterial community inhabiting shale rock: macroscopic micrograph (**A**) and scanning electron micrographs (**B**,**C**). Diversity of dominating (>1%) phyla (**D**), classes (**E**), and genera (**F**) identified in the bacterial community.

This study aimed to explore the biosynthesis of tetrapyrrole cofactors by a BC that inhabits fossil porphyrin-containing shale rock (Figure 2A–C). These porphyrins found in shale rock are known as geoporphyrins or sedimentary porphyrins. We conducted our research on Kupferschiefer shale rock located in Fore-Sudetic Monocline (SW Poland). It is a highly mineralized sedimentary Lopingian rock rich in fossil organic matter. Previous studies have confirmed the presence of iron, vanadyl, nickel, and cobalt geoporphyrins in Kupferschiefer shale rock (Table 1) [8,9,10,11]. 

This study is part of a project investigating the effects of bacteria on sedimentary geoporphyrins. Previous studies have shown that bacteria degrades or cleaves aliphatic and aromatic substituents of nickel and vanadyl geoporphyrins, leading to the formation of modified geoporphyrins, and also degrades the tetrapyrrole ring. The end products observed due to the microbial activity on shale rock are mono-, di-, and tripyrrole compounds [12]. Table 1 shows the list of modified geoporphyrins and pyrrole compounds identified in the shale rock inhabited by bacteria. Similar results were reported earlier by Grice et al. [13] who detected, for example, the presence of 1*H*-pyrrole-2,5-diones.

As part of this research, we investigated eight biosynthetic pathways, including the pathways of uroporphyrinogen III, siroheme, heme, and cobalamin (Figure 1). We analyzed three known biosynthetic pathways (protoporphyrin-, coproporphyrin-, and siroheme-dependent pathways) for heme. Furthermore, we divided the cobalamin biosynthetic pathways into three—aerobic and anaerobic biosynthesis of cob(II)yrinate a,c-diamide and biosynthesis of cobalamin from cob(II)yrinate a,c-diamide. Through metagenomic and metaproteomic analyses of BC, we identified protein-encoding genes (PEGs) and enzymatic proteins expressed as protein sequences (PSs) involved in the biosynthesis of tetrapyrrole cofactors. In addition, we searched for proteins containing the studied cofactors in the BC metaproteome. In the BC examined, we did not detect either PEGs or PSs that are involved in the F_430_ biosynthetic pathway.

## 2. Results

### 2.1. Taxonomic Diversity of BC Inhabiting Black Shale

With a total content of 82%, nearly five phyla of bacteria were found to be predominant in the investigated BC (Figure 2D). The dominant phyla observed were *Proteobacteria* (60.12%) and *Actinobacteria* (15.62%). *Bacteroidetes*, *Firmicutes*, and *Nitrospirae* were the other phyla with more than 1% dominance.

The dominant classes of bacteria were *Gammaproteobacteria* (33.85%), *Actinobacteria* (15.37%), *Alphaproteobacteria* (10.66%), and *Betaprotobacteria* (9.09%) (Figure 2E), while *Nitrospira* (1.69%) and *Bacilli* (1.28%) classes were found in smaller numbers. The other identified classes of bacteria accounted for less than 1% of the total.

In the analyzed BC, *Pseudomonas* was observed as the predominant genus, accounting for up to 20% of all the identified genera (Figure 2F).

### 2.2. General Characteristics of the Metagenome and Metaproteome of BC

**Figure 3:**
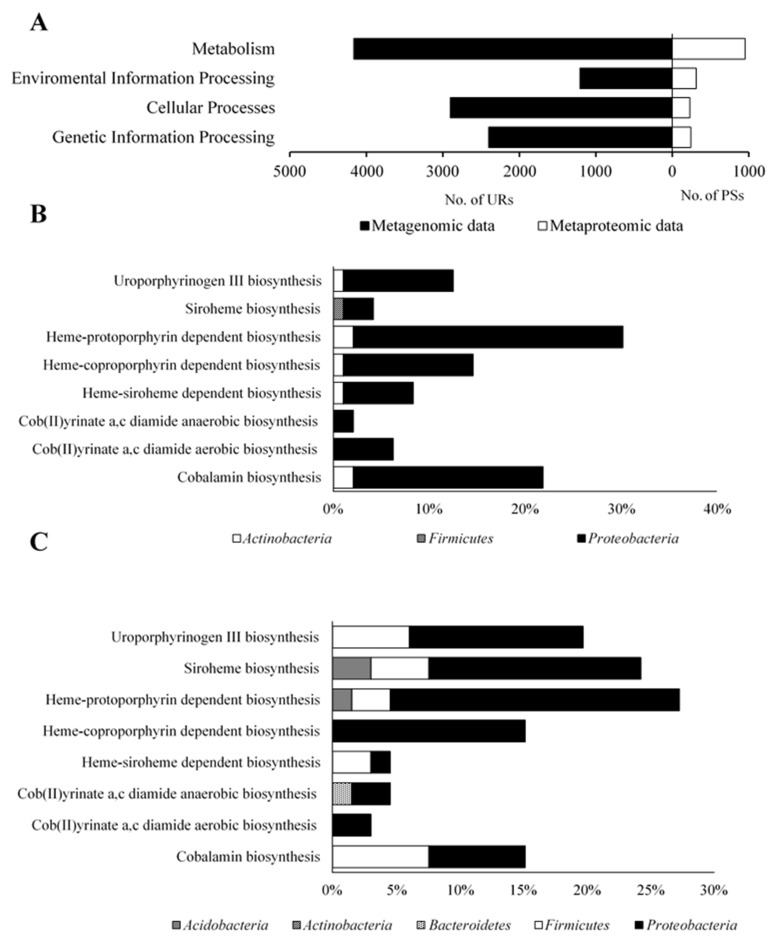
General characteristics of the metagenome and metaproteome of bacterial community. (**A**) The number of unique reads and protein sequences in the four main functional categories; taxonomic origin of the detected protein-encoding genes (**B**) and protein sequences (**C**) involved in biosynthesis of tetrapyrrole cofactors.

Metagenomic analysis of the studied BC showed that 99.6% of PEGs belonged to the *Bacteria* domain and the remaining PEGs (0.4%) belonged to *Archaea, Eukaryote*, and *Virus* domains (unpresented data). Metaproteomic analysis of BC showed that 100% of PSs belonged to the *Bacteria* domain (unpresented data). PEG-encoded enzymes involved in metabolism, environmental information processing, cellular processes, and genetic information processing accounted for 4166, 1208, 2901, and 2401 unique reads (URs), respectively (Figure 3A), whereas the metaproteome of BC consisted of 952, 311, 228, and 241 PSs that were involved in the mentioned functional categories, respectively. 

To estimate microbial richness at different taxonomic levels and to assess sample saturation for genes involved in porphyrin metabolism as a function of sequencing depth, rarefaction curves were generated (Figure S9). In both cases, the rarefaction curves reached a plateau, suggesting that sufficient sequencing saturation was achieved for taxonomic and genetic evaluation for the analyzed metabolic pathways. 

In the studied the BC metagenome analysis showed that 32 PEGs were involved in the major biosynthetic pathways of tetrapyrrole cofactor (Table S2*)*. Among them, the *Proteobacteria* URs accounted for 91.67%, *Actinobacteria* URs 7.29%, and *Firmicutes* URs 1.04% (Figure 3B). In addition, the metaproteomic analysis showed that 66 PSs participated in the biosynthesis of these compounds, of which the PSs of *Proteobacteria* (83.33%) and *Firmicutes* (10.61%) were predominant. *Acidobacteria*, *Actinobacteria*, and *Bacteroidetes* together accounted for the remaining 6.06% of PSs (Figure 3C, Table S3). Most of the URs and PSs identified in the studied BC were found to be involved in protoporphyrin-dependent heme metabolism (30.21% URs and 27.27% PSs) (Figure 3B,C).

### 2.3. Biosynthesis of Cofactors Detected in the Studied BC

#### 2.3.1. Uroporphyrinogen III Biosynthesis

**Figure 4:**
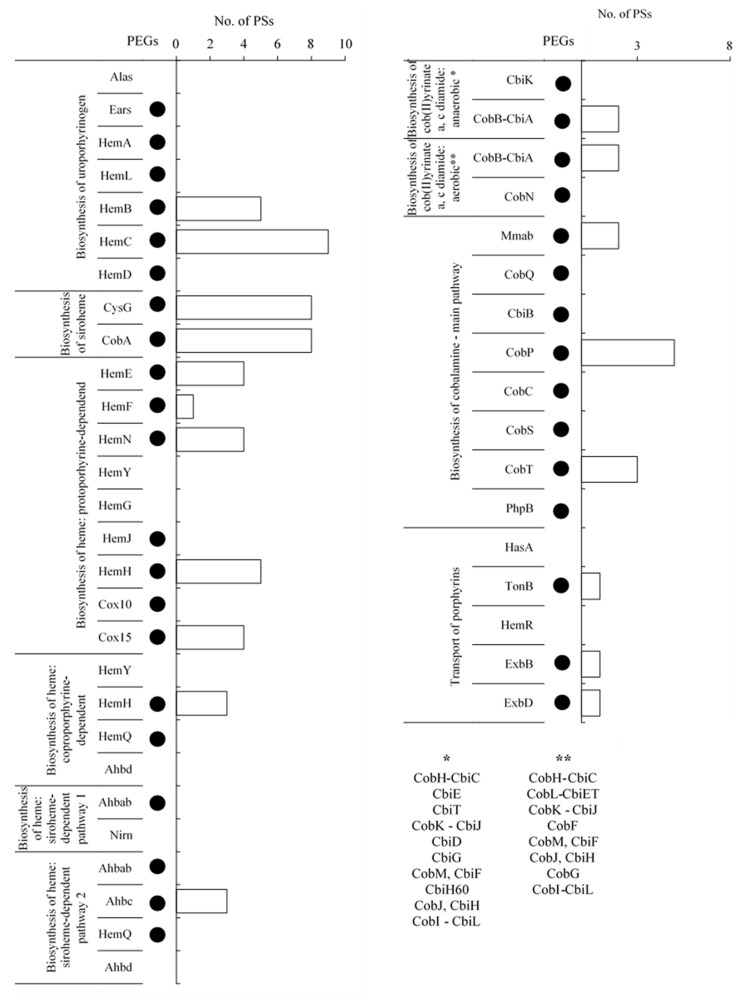
Detection of protein-encoding genes in the metagenome and number of protein sequences identified in the metaproteome of bacterial community involved in the biosynthesis of tetrapyrrole cofactors.* List of enzymes involved in anaerobic biosynthesis of cob(II)yrinate diamide detected neither in metagenome nor in metaproteome of bacterial community. ** List of enzymes involved in aerobic biosynthesis of cob(II)yrinate diamide detected neither in metagenome nor in metaproteome of bacterial community.

According to the literature data, the biosynthetic pathway of uroporphyrinogen III is depicted in Figure S1 [14,15,16]. Table S1 shows the lists of enzymes involved in this pathway. Figure 4 shows the results of the metagenomic and metaproteomic analyses of the tested BC.

Uroporphyrinogen III biosynthesis occurs with the use of Glycine or L-glutamate is the basic building block utilized in the biosynthesis of the pyrrole rings of uroporphyrinogen III. In the case of glycine substrate pathway, this compound is converted to 5-aminolevulinate by 5-aminolevulinate synthase (Alas) in a single step. L-glutamate is converted to 5-aminolevulinate by glutamyl-tRNA synthetase (Ears), glutamyl-tRNA reductase (HemA), and glutamate-1-semialdehyde 2,1-aminomutase (HemL). In the metagenome of BC, PEGs of all enzymes involved in the biosynthesis of uroporphyrinogen III using L-glutamate as the starting substrate were detected. However, no PSs were detected. The steps involved in the conversion of 5-aminolevulinate to uroporphyrinogen III are common to both pathways. 5-Aminolevulinate is transformed to porphobilinogen by porphobilinogen synthase (HemB), four molecules of which form a four-pyrrole hydroxymethylbilane compound catalyzed by hydroxymethylbilane synthase (HemC). The metagenomic analysis of the study showed the presence of PEGs of both enzymes. In addition, the metaproteomic analysis showed the presence of five and nine PSs that matched these two enzymes, respectively.

In the final reaction of the biosynthetic pathway, the tetrapyrrole ring is closed by uroporphyrinogen III synthase (HemD), resulting in the formation of uroporphyrinogen III. In the studied BC, the PEG of the HemD enzyme was observed, but the matching PSs were not detected.

#### 2.3.2. Siroheme Biosynthesis

According to the literature data, the biosynthetic pathway of siroheme is depicted in Figure S2 [17,18]. Table S1 shows the lists of enzymes involved in this pathway. Figure 4 shows the results of the metagenomic and metaproteomic analyses of the analyzed BC.

The step in which the heme and siroheme pathways are separated is the conversion of uroporphyrinogen III to precorrin 2. The biosynthesis of siroheme is a three-step process. The first step involves the formation of precorrin-2, in which the two methyl groups derived from S-adenosyl-L-methionine on carbons two and seven of uroporphyrinogen III are cleaved. The second step is the formation of sirohydrochlorin, which is a dehydrogenation (NAD^+^-dependent) process. The third step is the synthesis of siroheme, which involves the chelation of ferrous iron to sirohydrochlorin to form siroheme. The steps that follow are catalyzed by uroporphyrin III C-methyltransferase (CobA) or uroporphyrin III C-methyltransferase/precorrin-2 dehydrogenase/sirohydrochlorin ferrochelatase (CysG). The metagenomic analysis of the study showed the presence of PEGs of all enzymes, and metaproteomic analysis showed eight PSs matching both enzymes.

#### 2.3.3. Heme Biosynthesis

##### Protoporphyrin-Dependent Heme Biosynthetic Pathway

According to the literature data, the protoporphyrin-dependent heme synthesis pathway is depicted in Figure S3 [19,20]. Table S1 shows the lists of enzymes involved in this metabolic pathway. Figure 4 shows the results of the metagenomic and metaproteomic analyses of the analyzed BC.

The enzyme uroporphyrinogen decarboxylase (HemE) shortens the four substituents derived from propanoic acid to methyl substituents in uroporphyrinogen III, resulting in coproporphyrinogen III. The metagenome of BC analyzed in this study showed the presence of PEG of HemE and four PSs matching the enzyme. Coproporphyrinogen III is converted by enzymes coproporphyrinogen III oxidase (HemF) or oxygen-independent coproporphyrinogen III oxidase (HemN) to protoporphyrinogen IX by cleaving hydrogen from two propyl acid substituents and changing them to ethynyl substituents. The presence of PEGs of both enzymes and one PSs matching HemF and four PSs matching HemN were detected in analyses in the studied BC.

Further conversions of protoporphyrinogen IX to PPIX are catalyzed by three oxidases: protoporphyrinogen/coproporphyrinogen III oxidase (HemY), menaquinone-dependent protoporphyrinogen oxidase (HemG), and protoporphyrinogen IX oxidase (HemJ). In the BC metagenome, only PEGs of the latter enzyme HemJ were detected, whereas no PSs matching the enzyme HemJ were identified. Protoporphyrin/coproporphyrin ferrochelatase (HemH), a PPIX molecule, attaches iron as the central ion, resulting in the formation of protoheme (heme B), which is required in the synthesis of cytochrome B and can transform into different types of heme. The studied BC was found to have the PEG of HemH and five PSs matching this enzyme.

In the next steps, heme B is converted to heme O by heme O synthase (Cox10) and then to heme A by heme A synthase (Cox15). HemA is used as a cofactor of cytochrome A. The study results showed the presence of PEGs of both enzymes; however, four PSs matching only enzyme Cox15 were detected.

##### Coproporphyrin-Dependent Heme Biosynthetic Pathway

Coproporphyrin-dependent heme synthesis is an alternative heme biosynthetic pathway that uses part of the primary heme pathway [21]. This pathway is shown in Figure S4 based on the literature data [22,23,24]. Table S1 shows the lists of enzymes involved in this pathway. Figure 4 shows the results of the metagenomic and metaproteomic analyses.

In the abovementioned pathway, protoporphyrinogen/coproporphyrinogen III oxidase (HemY) converts coproporphyrinogen III to coproporphyrin III by removing hydrogen atoms from the pyrrole rings. However, the study results showed no PEGs and PSs of HemY in the investigated BC. Similar to the protoporphyrin-dependent heme biosynthetic pathway, protoporphyrin/coproporphyrin ferrochelatase (HemH) converts coproporphyrin III to Fe-coproporphyrin III by adding an iron atom. The results of this study showed PEG of HemH and five PSs of this enzyme in the investigated BC. Further, decarboxylation of Fe-coproporphyrin III substituents by hydrogen peroxide-dependent heme synthase (HemQ) or AdoMet-dependent heme synthase (Ahbd) results in the formation of heme B. The results showed PEG matching only HemQ enzyme in the studied BC, whereas no PSs matching the enzyme HemQ or Ahbd were detected.

##### Siroheme-Dependent Heme Biosynthetic Pathway

Another lesser known heme biosynthetic pathway is the siroheme pathway [21]. This pathway is depicted in Figure S5 based on the literature data [25,26,27]. Table S1 shows the list of enzymes involved in this pathway. Figure 4 shows the results of the metagenomic and metaproteomic analyses.

The first step in this pathway is the transformation of siroheme to heme B, in which two CO_2_ groups are separated by siroheme decarboxylase (Ahbab), resulting in the formation of 12,18-didecarboxysiroheme. The newly formed compound can transform to dihydro-heme d1 (the mechanism of this reaction has not been known yet) and then to heme d1 (Nirn) with the help of the enzyme dihydro-heme d1 dehydrogenase. In the investigated BC, only PEG of Ahbab was detected, but no PSs of any of these enzymes were found.

An alternative pathway for the synthesis of heme in this pathway is the conversion of 12,18-didecarboxysiroheme to Fe-coproporphyrin III catalyzed by Fe-coproporphyrin III synthase (Ahbc). In this reaction, two methanoic acid residues, present in the form of substituents, are removed from the porphyrin ring. The results showed PEG and three PSs matching Ahbc in the studied BC.

The final step is the decarboxylation of Fe-coproporphyrin III substituents to heme B by hydrogen peroxide-dependent heme synthase (HemQ) or AdoMet-dependent heme synthase (Ahbd). Metagenomic results of this study showed only PEG of HemQ; however, no PSs matching HemQ or Ahbd enzyme were detected.

#### 2.3.4. Cobalamin Biosynthesis

Cobalamin biosynthesis consists of two steps. Cob(II)yrinate a,c-diamide is synthesized in the first step, which is then converted to cobalamin [28]. The first step may take place under anaerobic or aerobic conditions.

##### Biosynthesis of Anaerobic and Aerobic Cob(II)yrinate a,c-Diamide

Anaerobic and aerobic synthesis of cob(II)yrinate a,c-diamide is shown in Figures S6 and S7, respectively, based on the literature data [28,29,30,31]. Table S1 shows the list of enzymes involved in these two pathways. Figure 4 shows the results of the BC metagenome and metaproteome analyses.

The results showed that only PEGs of CbiK and CobN were involved in anaerobic and aerobic pathways, respectively, and the metaproteome of BC had two PSs matching CobB-CbiA.

##### Biosynthesis of Cobalamin from Cob(II)yrinate a,c-Diamide

Biosynthesis of cobalamin from cob(II)yrinate a,c-diamide is shown in Figure S8 based on the literature data [28,32]. Table S1 shows the lists of enzymes involved in this pathway. Figure 4 shows the results of the metagenome and metaproteome analyses.

Cobalamin formation reactions can be carried out by using cob(II)yrinate a,c-diamide or cob(I)yrinate a, c diamide to form adenosyl cobyrinate a, c diamide. In both substrates, Co(III) in the corrinoid ring is reduced to Co(II) by transferring one electron. Furthermore, Co(II) is bound by corrinoid adenosyltransferase, resulting in the displacement of the lower axial substituent by an aromatic residue, as well as the transfer of the deoxyadenosyl group and oxidation of the cobalt atom to the Co(III) state. These reactions are carried out by cob(I)alamin adenosyltransferase (Mmab) in both the substrates. The results of this study showed the presence of PEG and two PSs matching Mmab.

In the next step, adenosylcobyrinic acid a,c-diamide is converted to adenosylcobyric acid by adenosylcobyric acid synthase (cobQ, cbiP), which catalyzes the four-step amidation sequence by forming triamide, tetraamide, and pentaamide as intermediates of adenosylcobyric acid. The PEG of CobQ was detected in this study, but the PS matching this enzyme was not found in the investigated BC.

The compound obtained is converted to adenosyl cobinamide by adenosylcobinamide-phosphate synthase (CbiB, CobD, CobC1). In this reaction, the lower-axis substituent of the NH group is cleaved, resulting in the formation of adenosyl cobinamide. The results of this study showed PEG of CbiB, but PS matching this enzyme was not detected in the BC.

Furthermore, adenosyl cobinamide is converted to adenosyl cobinamide phosphate and then to adenosylcobinamide-GDP by adenosylcobinamide-phosphate guanylyltransferase (CobP, CobU). An orthophosphoric acid residue is attached to the lower substituent in the first reaction, and adenosine is coupled to the attached phosphate in the second reaction. This study showed PEG of CobP and five PSs matching this enzyme.

In the final step, adenosylcobinamide-GDP is converted to cobalamin by adenosylcobinamide-GDP ribazoletransferase (CobS, CobV), which attaches alpha-ribazole to adenosylcobinamide-GDP molecule, resulting in the formation of the final product. This study showed the presence of PEG of CobS, but PS matching the enzyme CobS was not found.

An important part of this metabolic pathway is the formation of alpha-ribazole, which is added to adenosylcobinamide-GDP in the final step of cobalamin biosynthesis. Dimethylbenzimidazole is an important substrate that is converted to 5′-phosphate alpha-ribazole by nicotinate-nucleotide dimethylbenzimidazole phosphoribosyltransferase (CobU, CobT). This reaction attaches to the phospho-alpha-D-ribosyl substrate in place of the hydrogen present at the nitrogen atom. PEG and three PSs matching this enzyme were detected in the analyzed BC.

The resultant product is converted to alpha-ribazole, which is attached to the cortical ring. This reaction is catalyzed by adenosylcobalamin/alpha-ribazole phosphatase (PhpB) by cleaving the phosphoric acid residue from the substituent. The results showed PEG of PhpB, but PSs matching this enzyme were absent.

##### 2.3.5. Transport of Exogenous Porphyrins

In the BC examined, a transport system based on the TonB periplasmic protein with auxiliary heme acquisition protein (HasA), hemoglobin/transferrin/lactoferrin receptor protein (HemR), biopolymer protein transport ExbB, and biopolymer protein transport ExbD was detected (Figure 4). PEGs of TonB, ExbB, and ExbD were identified in the BC metagenome. Furthermore, one PS of tonB, ExbB, and ExbD was identified in the metaproteome (Figure 4).

#### 2.3.6. Tetrapyrrole Cofactor-Containing Enzymes Produced by BC

Table 2 lists the proteins containing tetrapyrrole cofactors in the BC metaproteome. Among these enzymes, (i) heme-containing cytochromes, peroxidases, and catalases; (ii) siroheme-containing sulfite reductases, and (iii) cobalamin-containing methyltrasferases and dehalogenases were detected.

## 3. Discussion

One of the basic elements of metagenome- and/or metaproteome-based studies of microbial metabolism is the characteristics of the tetrapyrrole cofactor biosynthesis [33,34,35]. However, most of the available literature data describe the biosynthesis of a single tetrapyrrole cofactor, with no detailed analysis of the biosynthesis of the four tetrapyrrole cofactors. Furthermore, a majority of the studies are limited to the analysis of single strain cultivated under laboratory conditions, and no data are available on the synthesis of cofactors by a community of microorganisms in their natural environment. There is also no mention of these metabolic processes with regard to the natural environment in which the microorganisms/communities are found and the potential use of chemical compounds—precursors—present in this environment. In this regard, our study is unique in terms of research material (BC), environment (sedimentary shale rock containing fossil tetrapyrroles), and research methodology. It is also worth emphasizing that the results of our previous geochemical studies of the environment complement the molecular analysis and provide a broader view of the processes under investigation.

The metagenomic and metaproteomic analyses of the tested BCs revealed the existence of tens of thousands of URs and hundreds of PSs involved in the basic metabolism (Figure 3A). Among them, 0.7% URs and 7.6% PSs were involved in the metabolism related to the biosynthesis of tetrapyrrole cofactors, the products of which are essential for many enzymatic reactions. Much higher results were obtained by Feng et al. [36], who employed the same method of analysis for examining the metagenome derived from soil from phosphate-producing chemical plants. Their study showed that approximately 4% of URs were involved in the biosynthesis of tetrapyrrole cofactors. Similar results were obtained by Ghosh and Das [37], who also used the KEGG base to perform a functional investigation of the microbiome from manganese-contaminated mine rocks.

The results of the present study showed that five of the eight metabolic pathways have a set of URs (uroporphyrinogen III, siroheme, protoporphyrin-dependent heme, siroheme-dependent heme, and cobalamin main biosynthetic pathways), including one set of enzymatic proteins (siroheme biosynthetic pathway) (Figure 4 and Figure S5). The remaining pathways lack at least one gene. For example, 66% of PEGs and 33% of PSs were identified in the coproporphyrin-dependent pathway of heme biosynthesis. However, proteins containing all of the examined cofactors were found in BC, and hemoproteins were particularly abundant (Table 2).

When interpreting the above results, one should consider the following questions: (1) do incomplete pathways result from difficulties in DNA isolation and insufficient depth of sequencing? (2) Why do bacteria produce main pathway enzymes if they do not synthesize the basic precursor, i.e., cob(II)yrinate a,c-diamide? (3) What is the role of enzymes that are identified in incomplete pathways? (4) What is the taxonomic origin of the identified PEGs and the relationship between the individual pathways? (5) How can the results obtained be linked to the geochemistry of the environment (shale rock) inhabited by BC?

The tested BC sample can be described as difficult, first of all in the context of DNA isolation. This is evidenced by the amount of sample used for DNA isolation in order to obtain the amount necessary for sequencing. On the other hand, the sequencing results (saturation of the readings) indicate the proper depth of sequencing (Figure S9). At the same time, it should be noted that similar results (incomplete pathways) were obtained for communities of marine bacteria, soil bacteria or even pure bacterial cultures, for which there were no difficulties with DNA isolation and sequencing [35,38,39,40,41].

For example, Doxey et al. [38] examined 430 metagenomes for the presence of genes encoding cobalamin and heme biosynthesis enzymes in marine *Thaumarchaeota*. No genes coding for: sirohydrochlorin cobaltochelatase, precorrin-6A/cobalt-precorrin-6A reductase, precorrin-3B synthase, precorrin-6A synthase, precorrin-6B C5,15-methyltransferase/cobalt-precorrin-6B C5,C15-methyltransferase, cobaltochelatase CobN participating in the aerobic and anaerobic synthesis of cobyrinate a, c-diamide were detected. The following genes were also not detected: cob(I)alamin adenosyltransferase, adenosylcobinamide kinase/adenosylcobinamide-phosphate guanylyltransferase, nicotinate-nucleotide—dimethylbenzimidazole phosphoribosyltransferase i alpha-ribazole phosphatase participating in the main pathway of cobalamin biosynthesis from cobyrinate a, c-diamide: Despite the lack of genes encoding the necessary enzymes for the biosynthesis of cobalamin, the biosynthesis of this compound takes place. Similar results were obtained for the heme biosynthesis. Only genes encoding enzymes responsible for the biosynthesis of uroporphyrinogen-III and converting heme B to heme H and O were discovered. 

In turn, Pan et al. [35] analyzed the metagenomic DNA of *Bathyarchaeota* found in mangrove sediments. Analysis of metagenomes revealed the presence of only about half of the genes encoding enzymes involved in cobalamin synthesis, including the cobalt chelatase gene, and only some of the genes encoding enzymes responsible for heme synthesis.

Similar results were also obtained by Panek and O′Brian [39], who studied the genomes of pure cultures of microorganisms, in which the genes encoding all enzymes of the heme biosynthetic pathway were also not detected, despite their ability to synthesize this cofactor. Among the examined genomes of bacteria, the gene encoding protoporphyrinogen IX oxidase was most often missing. This applies to strains belonging to *α-Proteobacteria*, e.g.,: *Agrobacterium tumefaciens, Brucella melitensis, Sinorhizobium meliloti,* *β-Proteobacteria*, e.g.,: *Neisseria meningitidis* (MC58 and Z2491), *Ralstonia solanacearum,* *σ*/ε-*Proteobacteria*, e.g.,: *Campylobacter jejuni, Helicobacter pylori* (26695 and J99), γ-*Proteobacteria*, e.g.,: *P. aeruginosa, Xylella fastidiosa.* The lack of the gene encoding protoporphyrinogen IX oxidase is also detected in heme biosynthesizing microorganisms that do not belong to the *Proteobacteria*, e.g., *Nostoc* sp. PCC 7120, *Synechocystis* PCC 6803. In addition, strains lacking the gene encoding protoporphyrinogen IX oxidase and uroporphyrinogen-III synthase were identified in α-*Proteobacteria*, e.g., *Caulobacter crescentus*, *Rickettsia conorii*, *R. prowazekii*.

Similar results were obtained by Glanville et al. [40], who in the genome of *P. aeruginosa* identified only selected genes encoding enzymes of the heme and cobalamin biosynthetic pathway.

According to Lu et al. [41], only about 10% of 155 soil microbial metagenomes contained complete cobalamin biosynthetic pathway. Similar results were obtained by Balabanova et al. [42], who found that the primary producers of cobalamin in the soil microbiome were bacteria of the genera *Proteobacteria*, *Actinobacteria*, *Firmicutes*, and *Nitrospirae*, which made up a small percentage of the community. The remaining microorganisms were only able to transport cobalamin.

In conclusion, as shown in the above studies, many bacteria do not have the complete heme biosynthetic pathways. The majority of the genomes examined lacked one or more gene-encoding enzymes involved in heme biosynthetic pathways. The same is true for cobalamin biosynthesis. 

One explanation for this phenomenon is that there are still undiscovered pathways in the biosynthesis of tetrapyrrole cofactors in bacteria [43]. However, incomplete pathways, including pathways in which microorganisms uptake readymade tetrapyrrole cofactors such as heme [44,45,46] and cobalamin [2,41,42] from the environment, have also been established. These studies have emphasized that the microorganisms transport cofactors from external sources. For example, living cells can discharge cobalamin into the soil or release it via cell lysis. Similarly in the case of heme, microorganisms secrete extracellular hemoproteins, e.g., extracellular peroxidases, which are then taken up by other microorganisms [47,48].

Furthermore, according to the literature data, the addition of certain chemical compounds to microbial medium increases the production of tetrapyrrole cofactors. For example, bacteria have been found to utilize PPIX as a source of the tetrapyrrole ring. These bacteria include, among others, bacteroids [49], *Lactococcus lactis* [50], and *Haemophilus influenzae* [51,52]. They have a shorter heme biosynthetic pathway that involves only ferrochelatase reactions, in which an iron atom is incorporated into the core of PPIX, thereby yielding heme. However, how PPIX are transported into the bacterial cell is still unknown, although it is believed that the transport of these compounds takes place via heme transport proteins [45]. Similarly, Jacobs et al. [53] found that adding coproporphyrinogen to the microbial medium increased the heme concentration generated by *P. fluorescens*, *P. denitrificans*, and *Escherichia coli*.

Similar conclusions can be drawn by analyzing cobalamin biosynthesis. In BC, we detected the entire biosynthetic pathway of this key compound from cob(II)yrinate a,c-diamide, with no enzymes involved in the process. According to a study by Lawrence et al. [54], cob(II)yrinate diamide may be extracted from the medium and converted to cobalamin. However, the role of CobB-CbiA in catalyzing the aerobic and anaerobic biosynthetic pathways of cob(II)yrinate a,c-diamide is worth mentioning. Analysis of the metagenome and metaproteome of BC showed that no genes and enzymes preceded or followed the reaction carried out by this enzyme. Two PSs of this enzyme were detected in the metaproteome. Various studies have shown that CobB-CbiA is utilized in the acetylation of proteins in the metabolism of microorganisms [55,56]. Because acetylated proteins are necessary for the normal functioning of metabolism in the cell, this enzyme can therefore be produced independently of cobalamin biosynthesis [57].

The literature data described above lead us to hypothesize whether bacteria can use organic compounds found in sedimentary rocks as tetrapyrrole precursors.

We obtained extremely important information with regard to this hypothesis from Demopoulos et al. [58] and Robinson [59], who showed that monopyrrole, dipyrrole, and tripyrroles compounds can be chemically converted into porphobilinogen and tetrapyrroles. Demopoulos et al. [58] showed the ability of pyrrole, pyrrole-3-acetic acid, 4-acylpyrrole-2-carboxyaldehydes, and pyrrole-2-carboxaldehyde to form porphobilinogen. In addition, Robinson [59] confirmed that uroporphyrinogen can be chemically synthesized from porphobilinogen (monopyrrole compounds) and 3-[5-[(*Z*)-[(5*Z*)-4-ethenyl-5-[(4-ethenyl-3-methyl-5-oxopyrrol-2-yl) methylidene]-3-methylpyrrol-2-ylidene]methyl]-2-hydroxy-4-methyl-1*H*-pyrrol-3-yl] propanoate (tripyrrole compounds). 

Taking into account the above information from the literature, it appears that the tested BC may also utilize pyrrole compounds formed as a result of geoporphyrin degradation. As mentioned, the Kupferschiefer shale contains a variety of geoporphyrins including cobalt protoporphyrin (IX) and pyrrole compounds that are formed as a result of bacterial degradation of sedimentary primary geoporphyrins (Table 1).

Finally, it is worth paying attention to the taxonomic origin, which indicates the dominant group of bacteria involved in the biosynthesis of tetrapyrrole cofactors. The results obtained with regard to the biosynthesis of tetrapyrroles revealed that *Proteobacteria* accounted for 91.67% of URs and 80.7% of PSs (Figure 3B,C). This is most probably related to the dominance of these organisms in BC, constituting more than 60% of the population (Figure 2D). Furthermore, *Proteobacteria* is one of the major phyla of bacteria characterized by its ability to synthesize cobalamin and iron porphyrins (heme, siroheme) [21,60]. Thus, the results obtained by us are consistent with the data obtained by other research teams.

To summarize, the metagenomic and metaproteomic analyses carried out in this study allowed us to gain a better understanding of the basic metabolism of tetrapyrrole cofactors by BC inhabiting the shale rock. Based on the results obtained, as well as on the literature data, one may consider the possibility of utilizing fossil primary or modified sedimentary porphyrins, as well as their bacterial degradation products, as precursors of tetrapyrrole cofactors. This hypothesis, of course, needs further evaluation, but its evidence would indicate an important and unique phenomenon of the tetrapyrrole ring cycle on Earth involving bacteria.

## 4. Materials and Methods

### 4.1. Site and Sample Description

Three BC-inhabited shale rock samples (Figure 2A–C) were aseptically collected at 32 °C and under 1080 hPa from a depth of approx. 800 m below sea level in the Lubin copper mine (KGHM Polska Miedź,Lubin, Poland). The pH of the sample was in the range of 8.2–8.4. Until laboratory processes, the collected samples were stored at −80 °C or −4 °C.

### 4.2. Isolation of DNA

DNA was isolated from the three BC samples according to the modified procedure of Zhou et al. [61]. Briefly, 100 g of sample was resuspended in 100 mL of DNA extraction buffer (0.1 M Na_2_EDTA, 0.1 M Tris–HCl, 0.1 M Na_2_HPO_4_, 1.5 M NaCl, 1% hexadecyltrimethylammonium bromide, pH 8.0) containing proteinase K and lysozyme (75 µL; 10 mg/mL) and was incubated overnight at 37 °C with horizontal shaking. Then, 20% sodium dodecyl sulfate was added, and the samples were incubated for 4 h at 65 °C and then centrifuged (6000 g, 10 min). The supernatants were harvested, mixed with an equal volume of chloroform/isoamyl alcohol mixture (24:1), and centrifuged again (6000 g, 10 min). After centrifugation, the aqueous phases were collected and precipitated overnight with 0.6 volume of isopropanol at room temperature. Next, the samples were centrifuged once again (16,000 g, 20 min, 4 °C), and the obtained pellets were washed with 70% cold ethanol and dried. Finally, DNA was isolated, resuspended in 50 µl of sterile deionized water, and stored at −80 °C until analysis.

### 4.3. Sequencing and Analysis of DNA

A barcoded library was prepared using the isolated DNA with Ion Xpress™ Plus Fragment Library Kit (Thermo Fisher, MD, USA) according to the manufacturer’s instructions. The library was then clonally amplified on Ion One Touch 2 system (Thermo Fisher) using Ion PI™ Template OT2 200 Kit v2 (Thermo Fisher) and sequenced on Ion Proton sequencer using Ion PI™ Sequencing 200 Kit v2 (Thermo Fisher) following the manufacturer’s instructions. The reads were demultiplexed with Torrent Suite software.

Qualitative preprocessing of the reads was performed with the Trimmomatic tool (v. 0.38) [62] (SLIDINGWINDOW:4:15, HEADCROP:3, CROP:250, and MINLEN:35). The quality of both pre- and postprocessed reads was assessed using FastQC (v. 0.11.08). Human reads were removed using BMTagger (v 1.1.0) [63] and human genome database GRCh38/hg38. Prior to the taxonomic analysis, artificial duplicate reads were removed using the k-mer approach. The obtained unassembled reads were assigned to the taxa using Kraken2 (v. 2.0.8-beta) [64] and the proteins of prokaryotic, fungal, and viral sequences of the NCBI RefSeq database (ftp://ftp.ncbi.nlm.nih.gov/refseq/, accessed on 7 November 2019. To perform functional analysis the reads were assembled using SPAdes (v.3.11.1) [65] with the “ion torrent” flag in the “careful” mode. Then, the assemblies were annotated using PROKKA (v. 1.13) [66] with default parameters and KofamScan (v. 1.3.0) [67] with KOfam HMM profiles (v. 26.04.2021). Hits having a bitsore of <60 and an e-value of >1e−5 were discarded. Based on the ko numbers and KEGG Orthology classification, the genes were assigned to metabolic pathways. The sequencing reads were realigned back to contigs using BBMap (v. 38.90) [68] with a “vslow” parameter. Duplicates were identified and removed using MarkDuplicates from the Picard tool kit. Only the BC sequences with unique best reads (URs) were taken into account, and duplicates were removed. The coverage for each predicted gene was normalized, expressed as transcripts per million [69], and averaged. Taxonomy for each gene was determined using Kraken2. Rarefaction curves for taxonomic analysis were generated using the krakefaction software (v.0.2.0) (https://github.com/phac-nml/krakefaction) and plotted as the number of unique phyla and genera as a function of sequencing depth. Rarefaction analysis carried out for complex genes that were assigned to the Porphyrin and chlorophyll metabolism pathway (KEGG Pathway: map00860) was performed using the RarefactionAnalyzer tool from the AMRPlusPlus pipeline [70] with 10 iterations per sampling level and 80 gene fraction threshold. The obtained data were plotted as the number of genes as a function of sequencing depth. The results of the metagenomic DNA sequencing are deposited at: http://www.ebi.ac.uk/ena/data/view/PRJEB21520 (ENA-FIRST-PUBLIC: accessed on 9 January 2019).

### 4.4. Isolation of Proteins

Proteins were extracted from the three BC samples as described by Ram et al. [71] Briefly, 100 g of sample was resuspended in 120 mL of 20 mM Tris–HCl (pH 8), shaken for 3 min, and sonicated on ice for up to 10 times for 1 min, with 1-min pauses in between (Sonics Vibracell; LABOPLUS, ModelCV18head, Sonics & Materials, CT, USA). The unlysed cells and fragments of the cell membrane were removed by adding 100 mL of 0.4 M Na_2_CO_3_ (pH 11) and centrifuging the suspension (6000 g, 20 min, 4 °C). The obtained supernatant was filtered through a 0.22-µm filter, and trichloroacetic acid was added at a ratio of 1:10 (*v*/*v*). The mixture was incubated overnight at 4 °C to allow for protein precipitation and then centrifuged again (20,000 g, 10 min, 4 °C). After centrifugation, the aqueous phase was discarded, and the pellet containing precipitated protein was resuspended in 0.5 mL of prechilled methanol (4 °C) and centrifuged once again (20,000 g, 10 min, 4 °C). The precipitate obtained was dried and stored at −80 °C. The sample preparation was performed in triplicate.

### 4.5. Identification of Proteins

Proteins were identified by liquid chromatography coupled to tandem MS (LC–MS-MS/MS) using nanoACQUITY (Waters, MA, USA) LC system and Orbitrap Velos mass spectrometer (Thermo Electron Corp., San Jose, CA, USA).

Identification was carried out at the Environmental Laboratory of Mass Spectrometry, Institute of Biophysics and Biochemistry (Polish Academy of Sciences, Warsaw, Poland). The equipment used for the analysis was sponsored in part by the Center for Preclinical Research and Technology (CePT), a project co-sponsored by the European Regional Development Fund and Innovative Economy, the National Cohesion Strategy of Poland.

Before identification, the proteins were subjected to a standard “in-solution digestion” procedure, during which they were reduced with 50 mM Tris(2-carboxyethyl)phosphine (60 min, 60 °C), alkylated with 200 mM *S*-methyl methanethiosulfonate (45 min, room temperature), and digested overnight with trypsin (Sequencing Grade Modified Trypsin; Promega V5111). The obtained peptide mixture was applied to an RP-18 precolumn (nanoACQUITY Symmetry^®^ C18; Waters 186003514), with water containing 0.1% trifluoroacetic acid as the mobile phase. Then, the mixture was transferred to a nano-HPLC RP-18 column (nanoACQUITY BEH C18; Waters 186003545) using an acetonitrile (ACN) gradient (5%–35% ACN in 180 min) with 0.05% formic acid (flow rate 250 µL/min). The column outlet was directly coupled to the ion source of the spectrometer, which was working in the regime of data-dependent MS to MS/MS switch. Each analysis was preceded by a blank run to rule out cross-contamination from previous samples.

The acquired raw data were processed by Mascot Distiller followed by Mascot Search (Matrix Science, London, UK; on-site license) against the NCBI protein database. Peptides having a Mascot score exceeding a threshold value corresponding to <5% expectation value, as calculated by the Mascot procedure, were considered as positively identified.

Metaproteomic analysis was performed according to Kanehisa et al. [72] using GhostKOALA automatic annotation and KEGG mapping service.

## Figures and Tables

**Table 1:** Primary and modified geoporphyrins and pyrrole-containing organic compounds detected in the Kupferschiefer shale rock.

Organic Compound	Name	Reference
**Primary** **geoporphyrins**	Iron aetioporphyrins (octaethyl porphyrins),	[8]
	Iron cycloalkanoporphyrins	
	Iron di-cycloalkanoporphyrins	
	Iron benz-cycloalkanoporphyrins	
	Vanadyl-cycloalkano-porphyrins	[9]
	Etio and DPEP iron porphyrins	[10]
	Etio,DPEP benzo-etio and benzo-DPEP vanadyl porphyrins	
	Vanadyl porphyrins of series etio/DPEP	[11]
	Octaethyl nickel porphyrin	[12]
	Octaethyl vanadyl porphyrin	
	Meso-tetraphenyl vanadyl porphyrin	
	Meso-tetraphenyl nickel porphyrin	
	Protoporphyrin IX cobalt	
**Modified geoporphyrins**	Diphenyl vanadyl porphyrin	[12]
	Tetraethyl vanadyl porphyrin	
	Vanadyl porphyrin	
	Tetraethyl nickel porphyrin	
	Nickel porphyrin	
**Organic compounds containing 3 pyrrole rings**	3-[2-[[3-(2-Carboxyethyl)-5-[(3.4-dimethyl-5-oxopyrrol-2-ylidene)methyl]-4-methyl-1H-pyrrol-2-yl]methylidene]-4-methyl-5-oxopyrrol-3-yl]propanoic acid	[12]
	3-[(5Z)-5-[[4-Ethenyl-5-[(Z)-(4-ethenyl-3-methyl-5-oxopyrrol-2-ylidene)methyl]-3-methyl-1H-pyrrol-2-yl]methylidene]-4-methyl-2-oxopyrrol-3-yl]propanoate	
**Organic compounds containing 2 pyrrole rings**	3,3′-Bipyrrole	
	2,2′-Bipyrrole	
	1,1′-Bipyrrole-2,2′,5,5′-tetraone	
	3,3′,4,4′-Tetramethyl-1H,1′H-2,2′-bipyrrole-5,5′-dicarboxylic acid	
**Organic compounds containing 1 pyrrole ring**	1H-Pyrrole	
	1H-Pyrrole-2-carboxylic acid	
	2H-Pyrrol-2-one, 5-[[2-[(4-aminophenyl)methylene]-3,4-dimethyll]methylene]-3-ethyl-1,5-dihydro-4-methyl	
	Indole acetic acid	
	1H-Indole-2-carboxylic acid	
	Indole carbaldehyde	
	1H-pyrrole-2,5-diones	[13]

**Table 2:** Enzymes containing tetrapyrrole cofactors detected in the studied metaproteome of bacterial community.

Enzyme	Accession	Score	Seq(sig)	emPAI	Genus/Species
Heme-Containing Cytochromes					
cbb3-type cytochrome c oxidase subunit ii	gi|653251020	62	1	0.19	*Dechloromonas agitata*
cb-type cytochrome c oxidase ccoo subunit	gi|4519209	55	1	0.12	*Magnetospirillum magnetotacticum*
cytochrome b6	gi|499245897	128	2	0.13	*Geobacter sulfurreducens*
cytochrome b6	gi|493924522	117	2	0.13	*Legionella drancourtii*
cytochrome bd-type quinol oxidase, subunit 1	gi|493975388	67	1	0.07	*Desulfovibrio magneticus*
cytochrome c	gi|504001995	71	2	0.1	*Azospira oryzae*
cytochrome c oxidase cbb3-type, subunit iii	gi|330949201	56	1	0.77	*Pseudomonas syringa. 1704B*
cytochrome c1	gi|268584477	58	1	0.11	*Neisseria gonorrhoeae* PID18
cytochrome cbb3	gi|499630345	55	1	0.27	*Thiobacillus denitrificans*
cytochrome d ubiquinol oxidase	gi|498185039	55	1	0.06	*Lactobacillus acidipiscis*
cytochrome o ubiquinol oxidase	gi|657198419	40	1	0.12	*Aeromonas caviae*
cytochrome p450	gi|503190589	20	1	0.1	*Frankia* sp. EuI1c
cytochrome p450	gi|664433299	44	1	0.07	*Streptomyces* sp. NRRL F-5140
cytochrome soxa	gi|499630342	44	1	0.11	*Thiobacillus denitrificans*
cytochrome soxa	gi|517333069	74	1	0.11	*Thiobacillus thioparu*
multispecies: cytochrome c	gi|494962102	68	1	0.25	*Sphingobium* sp.
flavocytochrome c sulfide dehydrogenase	gi|519012058	39	1	0.07	*Methylotenera* sp.
succinate dehydrogenase cytochrome b-556 subunit	gi|254672872	66	1	0.39	*Neisseria meningitidis alpha275*
thiosulfate reductase cytochrome b subunit	gi|488713780	61	1	0.15	*Myxococcus* sp.
**Heme-Containing Catalases**					
catalase	gi|491324047	515	11	0.84	*Acinetobacter* sp.CIP 53.82
catalase	gi|3927890	86	1	0.06	*Desulfovibrio vulgaris*
catalase	gi|520401	60	1	0.06	*Haemophilus influenzae*
catalase	gi|500251659	103	2	0.13	*Pseudomonas stutzeri*
catalase	gi|504938131	67	1	0.09	*Synechococcus* sp. PCC 6312
**Heme-Containing Peroxidases**					
hydroperoxidase	gi|647531474	46	1	0.04	*Shewanella marina*
hydroperoxidase II	gi|489375670	84	2	0.09	*Pseudomonas stutzeri*
hydroperoxidase II	gi|515815228	105	2	0.09	*Pseudomonas stutzeri*
peroxidase	gi|491142120	58	1	0.14	*Nitrococcus mobilis*
**Sirroheme-Containing Enzymes**					
nitrite reductase (NAD(P)H) large subunit	gi|159882975	62	1	0.04	*Hydrogenivirga* sp. 128-5-R1-1
nitrite reductase	gi|491129994	58	1	0.08	*Streptomyces ghanaensis*
nitrite reductase	gi|23392987	65	1	0.14	uncultured bacterium
sulfite reductase	gi|521065095	689	6	0.47	*Thiothrix disciformis*
sulfite reductase	gi|655041650	53	1	0.08	*Thiothrix lacustris*
sulfite reductase	gi|488797739	2076	7	1.18	*Thiothrix nivea*
sulfite reductase	gi|488797740	251	3	0.27	*Thiothrix nivea*
dissimilatory sulfite reductase alpha subunit	gi|30525497	52	1	0.08	uncultured sulfate-reducing bacterium
reverse-type dissimilatory siroheme sulfite reductase subunit A	gi|162072844	2187	7	1.3	*Thiothrix nivea* DSM 5205
reverse-type dissimilatory sulfite reductase (rDSR), alpha subunit (DsrA)	gi|385763698	48	1	0.07	uncultured bacterium 172H5
**Cobalamin-Containing Enzymes**					
methylcrotonoyl-CoA carboxylase	gi|648618195	36	1	0.06	*Niabella aurantiaca*
methylmalonyl-CoA carboxyltransferase	gi|587641191	40	1	0.06	*Skermanella stibiiresistens* SB22
methionyl-tRNA synthetase	gi|516410008	68	1	0.04	*Erwinia toletana*
methionyl-tRNA synthetase	gi|588476233	35	1	0.04	*Lactobacillus composti* JCM 14202

## Data Availability

The data presented in this study are available in Supplementary Materials.

## Supplementary Materials

The following are available online. Figure S1. Biosynthetic pathway of uroporphyrinogen III. Figure S2. Biosynthetic pathway of siroheme. Figure S3. Biosynthetic pathway of protoporphyrin-dependent heme. Figure S4. Biosynthetic pathway of coproporphyrin -dependent heme. Figure S5. Biosynthetic pathway of siroheme-dependent heme. Figure S6. Biosynthetic pathway of anaerobic cob(II)yrinate a,c-diamide. Figure S7. Biosynthetic pathway of aerobic cob(II)yrinate a,c-diamide. Figure S8. Biosynthetic pathway of cobalamin from cob(II)yrinate a,c-diamide. Figure S9. Rarefaction curves representing the relationship between the number of taxa (**A**) and genes (**B**) as a function of sequencing depth (proportion of reads sampled). Rarefaction analysis showed that sequencing saturation was achieved in both cases. Table S1. Metabolism of tetrapyrrole cofactors: list of enzymes involved in the biosynthesis of siroheme, heme, and cobalamin [73,74,75,76,77,78,79,80,81,82,83,84,85,86,87,88,89,90,91,92,93]. Table S2. List of protein-encoding genes detected in the metagenome of bacterial community involved in biosynthetic pathways under study. Table S3. List of protein sequences detected in the metaproteome of bacterial community involved in biosynthetic pathways under study.

## Sample Availability

Not available.
