# Supplementary material for: Biosynthesis of Tetrapyrrole Cofactors by Bacterial Community Inhabiting Porphyrine-Containing Shale Rock (Fore-Sudetic Monocline)
Source: Molecules. 2021 Nov 8;26(21):6746. doi: 10.3390/molecules26216746 (PMC8587615; doi:10.3390/molecules26216746)
Supplement: Supplementary file 1 [file molecules-26-06746-s001.zip › Supplementary Material Figure S9. Supplementary information of the studied metagenome - rarefaction curves.pdf]

Supplementary information of the studied metagenome - rarefaction curves

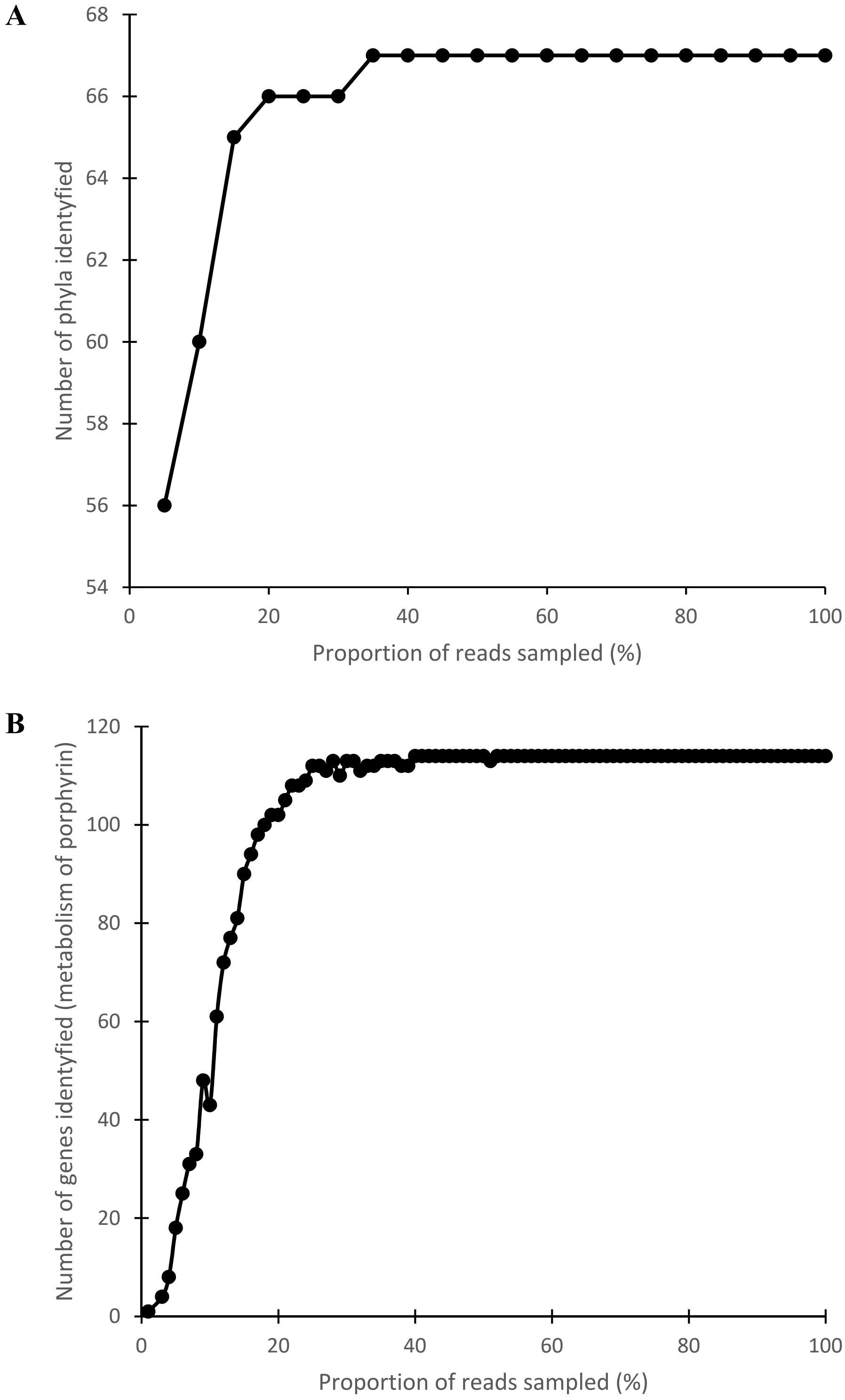

Figure S9. Rarefaction curves representing the relationship between the number of taxa (A) and genes (B) as a function of sequencing depth (proportion of reads sampled). Rarefaction analysis showed that sequencing saturation was achieved in both cases.
