# Supplementary material for: Biosynthesis of Tetrapyrrole Cofactors by Bacterial Community Inhabiting Porphyrine-Containing Shale Rock (Fore-Sudetic Monocline)
Source: Molecules. 2021 Nov 8;26(21):6746. doi: 10.3390/molecules26216746 (PMC8587615; doi:10.3390/molecules26216746)
Supplement: Supplementary file 1 [file molecules-26-06746-s001.zip › Supplementary Material Table S1. Figure S1-S8. Supplementary information of the studied biosynthetic pathways of tetrapyrrole cofactors.pdf]

Supplementary information about studied pathways of tetrapyrrole cofactors biosynthesis

Table S1. Metabolism of tetrapyrrole cofactors: list of enzymes involved in the biosynthesis of siroheme, heme and cobalamin

Name of enzyme	EC number	Gene shortcut	Reaction	References
**Uroporphyrinogen III biosynthesis pathway**				
5-aminolevulinate biosynthesis, pathway 1				
5-aminolevulinate synthase	2.3.1.37	ALAS	succinyl-CoA + glycine = 5-aminolevulinate + CoA + CO2	[14]
5-aminolevulinate biosynthesis, pathway 2				
glutamyl-tRNA synthetase	6.1.1.17	EARS, gltX	ATP + L-glutamate + tRNAGlu = AMP + diphosphate + L-glutamyl-tRNAGlu	[73]
glutamyl-tRNA reductase	1.2.1.70	hemA	L-glutamate 1-semialdehyde + NADP+ + tRNAGlu = L-glutamyl-tRNAGlu + NADPH + H+	[16]
glutamate-1-semialdehyde 2,1-aminomutase	5.4.3.8	hemL	L-glutamate 1-semialdehyde = 5-aminolevulinate	[74]
main pathway of uroporphyrinogen III biosynthesis				
porphobilinogen synthase	4.2.1.24	hemB, ALAD	2 5-aminolevulinate = porphobilinogen + 2 H2O	[75,76]
hydroxymethylbilane synthase	2.5.1.61	hemC, HMBS	4 porphobilinogen + H2O = hydroxymethylbilane + 4 NH3	[15,77]
uroporphyrinogen-III synthase	4.2.1.75	hemD, UROS	hydroxymethylbilane = uroporphyrinogen III + H2O	[18]
**Siroheme biosynthesis pathway**				
uroporphyrin-III C-methyltransferase	2.1.1.107	cobA, cobA-hemD, MET1, hemX	2 S-adenosyl-L-methionine + uroporphyrinogen III = 2 S-adenosyl-L-homocysteine + precorrin-2	[18]
uroporphyrin-III C-methyltransferase / precorrin-2 dehydrogenase / sirohydrochlorin ferrochelatase	2.1.1.107; 1.3.1.76 4.99.1.4	cysG, MET8	precorrin-2 + NAD+ = sirohydrochlorin + NADH + H+	[17]
**Protoporphyrin-dependent heme biosynthesis pathway**				
uroporphyrinogen decarboxylase	4.1.1.37	hemE, UROD	uroporphyrinogen III = coproporphyrinogen III + 4 CO2	[19,21,22]
coproporphyrinogen III oxidase	1.3.3.3	CPOX, hemF	coproporphyrinogen III + O2 + 2 H+ = protoporphyrinogen-IX + 2 CO2 + 2 H2O	[19,78]
oxygen-independent coproporphyrinogen III oxidase	1.3.98.3	hemN, hemZ	coproporphyrinogen III + 2 S-adenosyl-L-methionine = protoporphyrinogen IX + 2 CO2 + 2 L-methionine + 2 5'-deoxyadenosine	[20]
protoporphyrinogen/ coproporphyrinogen III oxidase	1.3.3.4; 1.3.3.15	PPOX, hemY	protoporphyrinogen IX + 3 O2 = protoporphyrin IX + 3 H2O2	[21,23]
menaquinone-dependent protoporphyrinogen oxidase	1.3.5.3	hemG	protoporphyrinogen IX + 3 quinone = protoporphyrin IX + 3 quinol	[79,80]

protoporphyrinogen IX oxidase	1.3.99.-	hemJ	protoporphyrinogen IX + 3 acceptor = protoporphyrin + 3 reduced acceptor	[81]
protoporphyrin/coproporphyrin ferrochelatase	4.99.1.1; 4.99.1.9	hemH, FECH	protoheme + 2 H+ = protoporphyrin + Fe2+	[82]
heme o synthase	2.5.1.141	COX10, ctaB, cyoE	(2E,6E)-farnesyl diphosphate + protoheme IX + H2O = diphosphate + ferroheme o	[83]
heme a synthase	1.17.99.9	COX15, ctaA	ferroheme o + H2O + 2 acceptor = ferroheme a + 2 reduced acceptor	[84]
**Coproporphyrin -dependent heme biosynthesis pathway**				
protoporphyrinogen/ coproporphyrinogen III oxidase	1.3.3.4; 1.3.3.15	PPOX, hemY	coproporphyrinogen III + 3 O2 = coproporphyrin III + 3 H2O2	[21,23]
protoporphyrin/coproporphyrin ferrochelatase	4.99.1.1; 4.99.1.9	hemH, FECH	Fe-coproporphyrin III + 2 H+ = protoporphyrin + Fe2+	[82]
hydrogen peroxide-dependent heme synthase	1.3.98.5	hemQ	Fe-coproporphyrin III + 2 H2O2 = protoheme + 2 CO2 + 4 H2O	[27]
AdoMet-dependent heme synthase	1.3.98.6	ahbD	Fe-coproporphyrin III + 2 S-adenosyl-L-methionine = protoheme + 2 CO2 + 2 5'-deoxyadenosine + 2 L-methionine	[24]
**Siroheme-dependent heme biosynthesis pathway**				
Pathway I				
siroheme decarboxylase	4.1.1.111	ahbAB	siroheme = 12,18-didecarboxysiroheme + 2 CO2	[26]
dihydro-heme d1 dehydrogenase	-	NirN	dihydro-heme d1 + acceptor = heme d1 + reduced acceptor	[25]
Pathway II				
siroheme decarboxylase	4.1.1.111	ahbAB	siroheme = 12,18-didecarboxysiroheme + 2 CO2	[26]
Fe-coproporphyrin III synthase	-	Ahbc	12,18-didecarboxysiroheme = Fe-coproporphyrin III	
hydrogen peroxide-dependent heme synthase	1.3.98.5	hemQ	Fe-coproporphyrin III + 2 H2O2 = protoheme + 2 CO2 + 4 H2O	[27]
AdoMet-dependent heme synthase	1.3.98.6	ahbD	Fe-coproporphyrin III + 2 S-adenosyl-L-methionine = protoheme + 2 CO2 + 2 5'-deoxyadenosine + 2 L-methionine	[24]
**Anaerobic cob(II)yrinate a, c diamide biosynthesis pathway**				
sirohydrochlorin cobaltochelatase	4.99.1.3	cbiK	cobalt-sirohydrochlorin + 2 H+ = sirohydrochlorin + Co2+	[31]
precorrin-2/cobalt-factor-2 C20-methyltransferase	2.1.1.130; 2.1.1.151	cobI - cbiL	S-adenosyl-L-methionine + cobalt-factor II = S-adenosyl-L-homocysteine + cobalt-factor III	[85]
precorrin-3B C17-methyltransferase	2.1.1.131	cobJ, cbiH	S-adenosyl-L-methionine + precorrin-3B = S-adenosyl-L-homocysteine + precorrin-4	[86]
cobalt-factor III methyltransferase	2.1.1.272	cbiH60	S-adenosyl-L-methionine + cobalt-factor III + reduced acceptor = S-adenosyl-L-homocysteine + cobalt-precorrin-4 + acceptor	[87]
precorrin-4/cobalt-precorrin-4 C11-methyltransferase	2.1.1.133 2.1.1.271	cobM, cbiF	S-adenosyl-L-methionine + cobalt-precorrin-4 = S-adenosyl-L-homocysteine + cobalt-precorrin-5A	[29]

cobalt-precorrin 5A hydrolase	3.7.1.12	cbiG	cobalt-precorrin-5A + H_2O = cobalt-precorrin-5B + acetaldehyde + 2 H^+	[87]
cobalt-precorrin-5B (C1)-methyltransferase	2.1.1.19	cbiD	S-adenosyl-L-methionine + cobalt-precorrin-5B = S-adenosyl-L-homocysteine + cobalt-precorrin-6A	
precorrin-6A/cobalt-precorrin-6A reductase	1.3.1.54; 1.3.1.106	cobK - cbiJ	cobalt-precorrin-6B + NAD^+ = cobalt-precorrin-6A + NADH + H^+	
cobalt-precorrin-6B (C15)-methyltransferase	2.1.1.196	cbiT	S-adenosyl-L-methionine + cobalt-precorrin-6B = S-adenosyl-L-homocysteine + cobalt-precorrin-7 + CO_2	
cobalt-precorrin-7 (C5)-methyltransferase	2.1.1.289	cbiE	S-adenosyl-L-methionine + cobalt-precorrin-7 = S-adenosyl-L-homocysteine + cobalt-precorrin-8	[28]
precorrin-8X/cobalt-precorrin-8 methylmutase	5.4.99.61; 5.4.99.60	cobH-cbiC	cobalt-precorrin-8 = cobyrinate	[30]
cobyrinic acid a,c-diamide synthase	6.3.5.9; 6.3.5.11	cobB-cbiA	2 ATP + cobyrinate + 2 L-glutamine + 2 H_2O = 2 ADP + 2 phosphate + cobyrinate a,c-diamide + 2 L-glutamate	[88]
**Aerobic cob(II)yrinate a, c diamide biosynthesis pathway**				
precorrin-2/cobalt-factor-2 C20-methyltransferase	2.1.1.130; 2.1.1.151	cobI - cbiL	S-adenosyl-L-methionine + precorrin-2 = S-adenosyl-L-homocysteine + precorrin-3A	[89]
precorrin-3B synthase	1.14.13.83	cobG	precorrin-3A + NADH + H^+ + O_2 = precorrin-3B + NAD^+ + H_2O	[28]
precorrin-3B C17-methyltransferase	2.1.1.13	cobJ, cbiH	S-adenosyl-L-methionine + precorrin-3B = S-adenosyl-L-homocysteine + precorrin-4	[86]
precorrin-4/cobalt-precorrin-4 C11-methyltransferase	2.1.1.133; 2.1.1.271	cobM, cbiF	S-adenosyl-L-methionine + precorrin-4 = S-adenosyl-L-homocysteine + precorrin-5	[29]
precorrin-6A synthase	2.1.1.152	cobF	S-adenosyl-L-methionine + precorrin-5 + H_2O = S-adenosyl-L-homocysteine + precorrin-6A + acetate	[28]
precorrin-6A/cobalt-precorrin-6A reductase	1.3.1.54; 1.3.1.106	cobK - cbiJ	precorrin-6B + $NADP^+$ = precorrin-6A + NADPH + H^+	
precorrin-6B C5,15-methyltransferase / cobalt-precorrin-6B C5,C15-methyltransferase	2.1.1.132; 2.1.1.289; 2.1.1.196	cobL-cbiET	2 S-adenosyl-L-methionine + precorrin-6B = 2 S-adenosyl-L-homocysteine + precorrin-8X + CO_2	[90]
precorrin-8X/cobalt-precorrin-8 methylmutase	5.4.99.61; 5.4.99.60	cobH-cbiC	precorrin-8X = hydrogenobyrinate	[91]
cobyrinic acid a,c-diamide synthase	6.3.5.9; 6.3.5.11	cobB-cbiA	2 ATP + hydrogenobyrinic acid + 2 L-glutamine + 2 H_2O = 2 ADP + 2 phosphate + hydrogenobyrinic acid a,c-diamide + 2 L-glutamate	[28]
cobaltochelatase CobN	6.6.1.2	cobN	ATP + hydrogenobyrinate a,c-diamide + Co^{2+} + H_2O = ADP + phosphate + cob(II)yrinate a,c-diamide + H^+	
**Cobalamin biosynthesis pathway from cob(II)yrinate a,c diamide**				

cob(I)alamin adenosyltransferase	2.5.1.17	MMAB, pduO, cobA, btuR	(1) 2 ATP + 2 cob(II)alamin + a reduced flavoprotein = 2 triphosphate + 2 adenosylcob(III)alamin + an oxidized flavoprotein;<br>(1a) 2 cob(II)alamin + 2 [corrinoid adenosyltransferase] = 2 [corrinoid adenosyltransferase]-cob(II)alamin;<br>(1b) a reduced flavoprotein + 2 [corrinoid adenosyltransferase]-cob(II)alamin = an oxidized flavoprotein + 2 [corrinoid adenosyltransferase]-cob(I)alamin (spontaneous);<br>(1c) 2 ATP + 2 [corrinoid adenosyltransferase]-cob(I)alamin = 2 triphosphate + 2 adenosylcob(III)alamin + 2 [corrinoid adenosyltransferase]	[32]
adenosylcobyric acid synthase	6.3.5.10	cobQ, cbiP	4 ATP + adenosylcobyrinic acid a,c-diamide + 4 L-glutamine + 4 H2O = 4 ADP + 4 phosphate + adenosylcobyric acid + 4 L-glutamate	[28]
adenosylcobinamide-phosphate synthase	6.3.1.10	cbiB, cobD	ATP + adenosylcobyric acid + (R)-1-aminopropan-2-ol = ADP + phosphate + adenosylcobinamide	
adenosylcobinamide kinase / adenosylcobinamide-phosphate guanylyltransferase	2.7.1.156; 2.7.7.62	cobP, cobU	RTP + adenosylcobinamide = adenosylcobinamide phosphate + RDP [where RTP is either ATP or GTP	
cobalamin biosynthesis protein CobC/ adenosylcobinamide-phosphate synthase	-	CobC	ATP + adenosylcobyric acid + (R)-1-aminopropan-2-ol = ADP + phosphate + adenosylcobinamide	
adenosylcobinamide kinase / adenosylcobinamide-phosphate guanylyltransferase	2.7.1.156; 2.7.7.62	cobP, cobU	GTP + adenosylcobinamide phosphate = diphosphate + adenosylcobinamide-GDP	[92]
adenosylcobinamide-GDP ribazoletransferase	2.7.8.26	cobS, cobV	(1) adenosylcobinamide-GDP + alpha-ribazole = GMP + adenosylcobalamin<br>(2) adenosylcobinamide-GDP + alpha-ribazole 5'-phosphate = GMP + adenosylcobalamin 5'-phosphate]	
nicotinate-nucleotide--dimethylbenzimidazole phosphoribosyltransferase	2.4.2.21	cobU, cobT	beta-nicotinate D-ribonucleotide + 5,6-dimethylbenzimidazole = nicotinate + alpha-ribazole 5'-phosphate	
alpha-ribazole phosphatase	3.1.3.73	cobC, phpB	(1) adenosylcobalamin 5'-phosphate + H2O = coenzyme B12 + phosphate<br>(2) alpha-ribazole 5'-phosphate + H2O = alpha-ribazole + phosphate	[93]

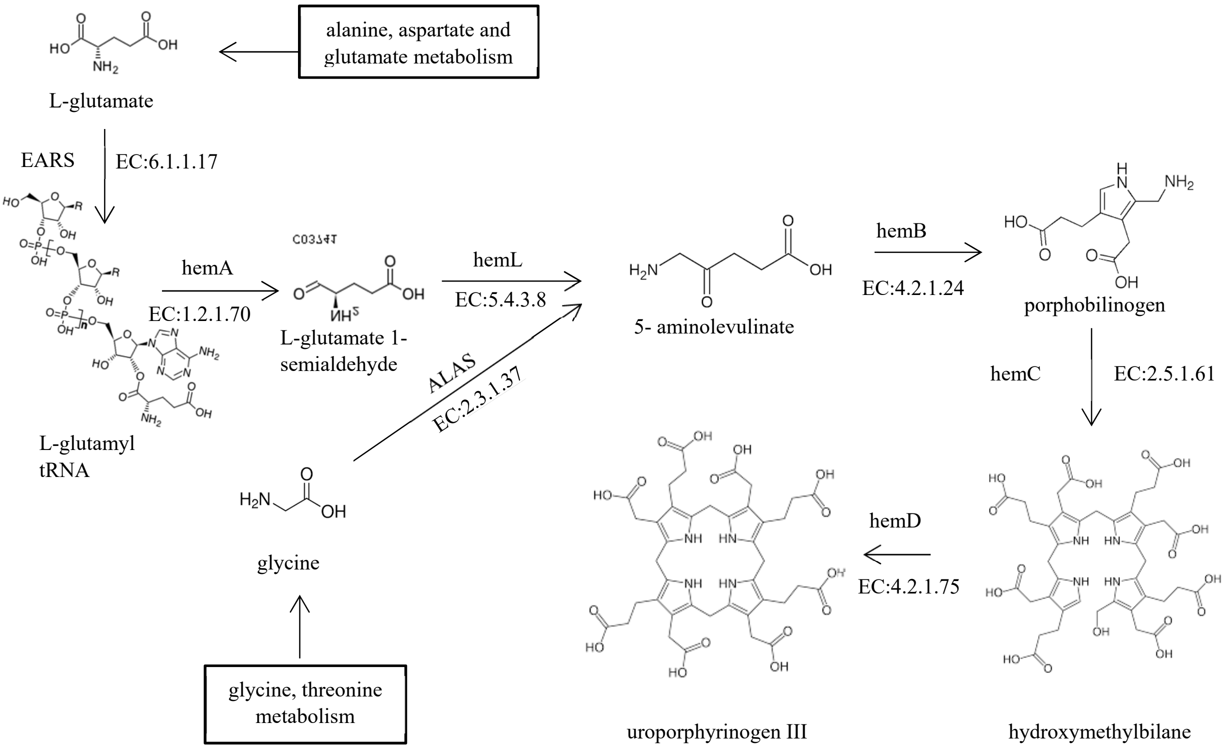

Figure S1. Biosynthetic pathway of uroporphyrinogen III

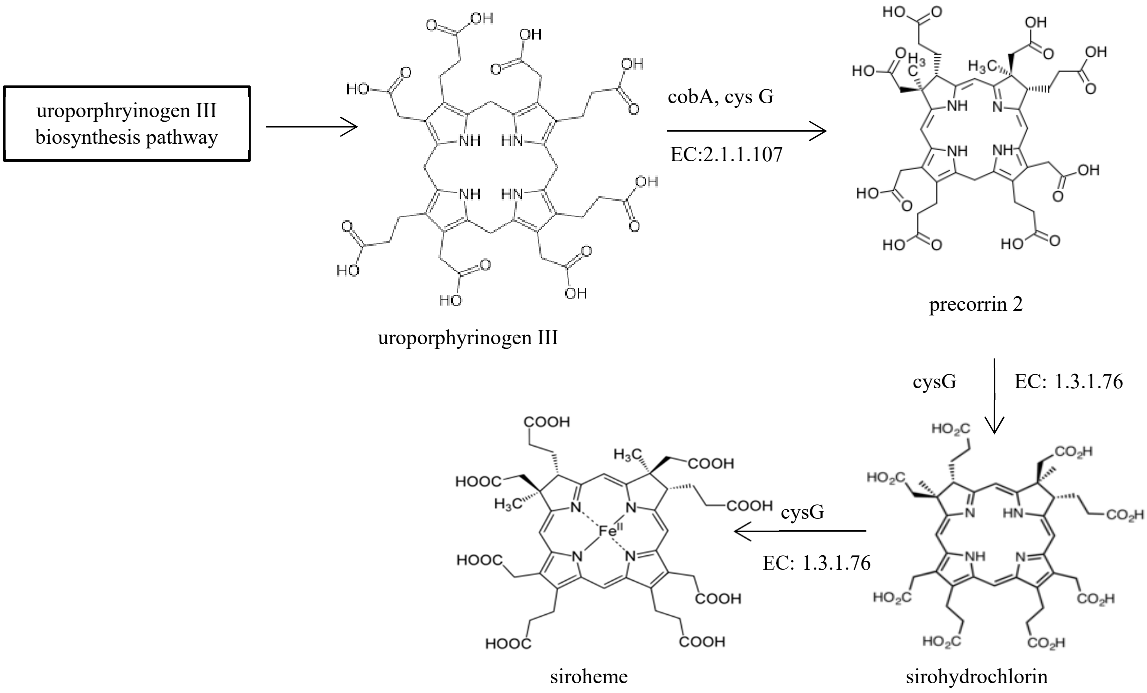

Figure S2. Biosynthetic pathway of siroheme

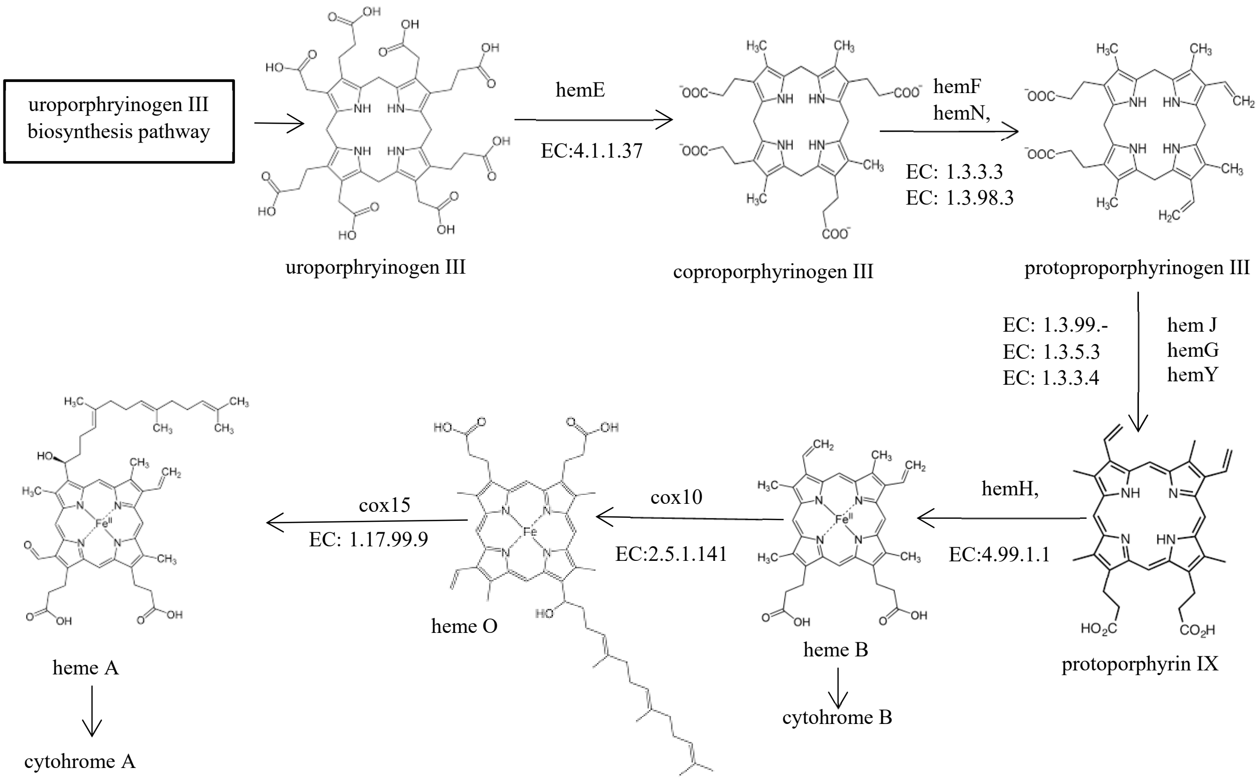

Figure S3. Biosynthetic pathway of protoporphyrin-dependent heme

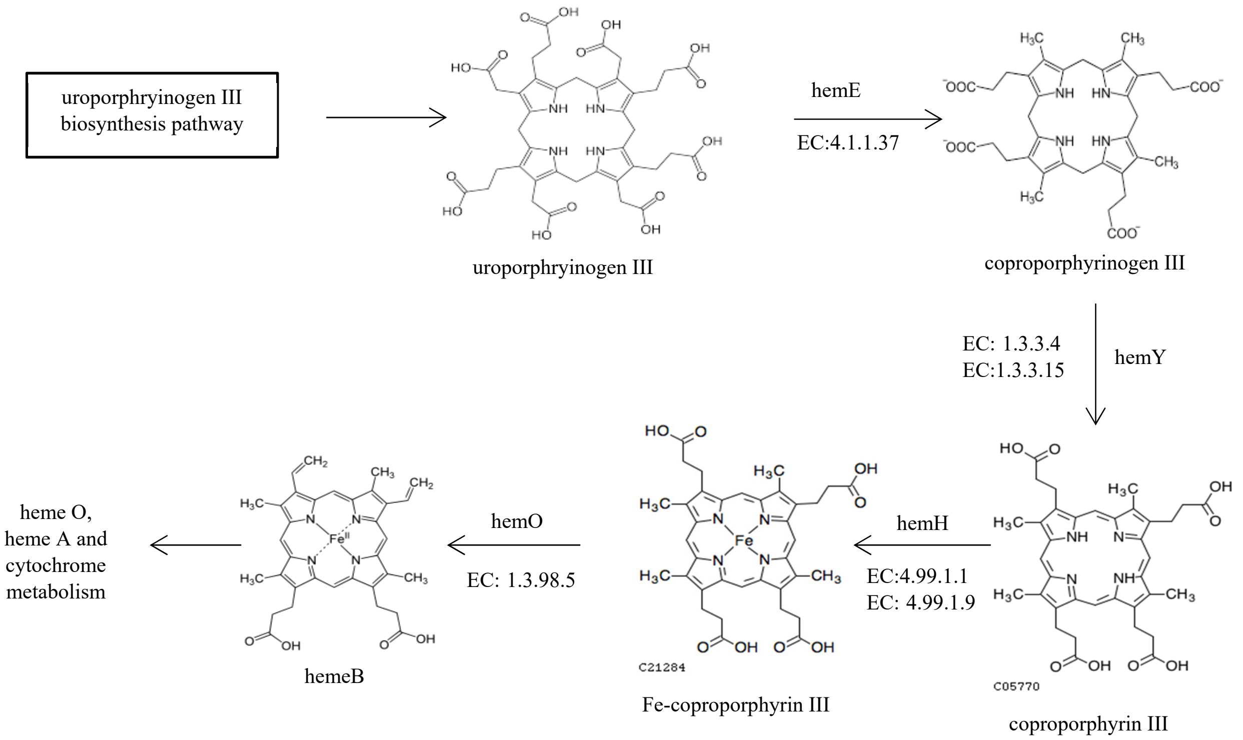

Figure S4. Biosynthetic pathway of coproporphyrin-dependent heme

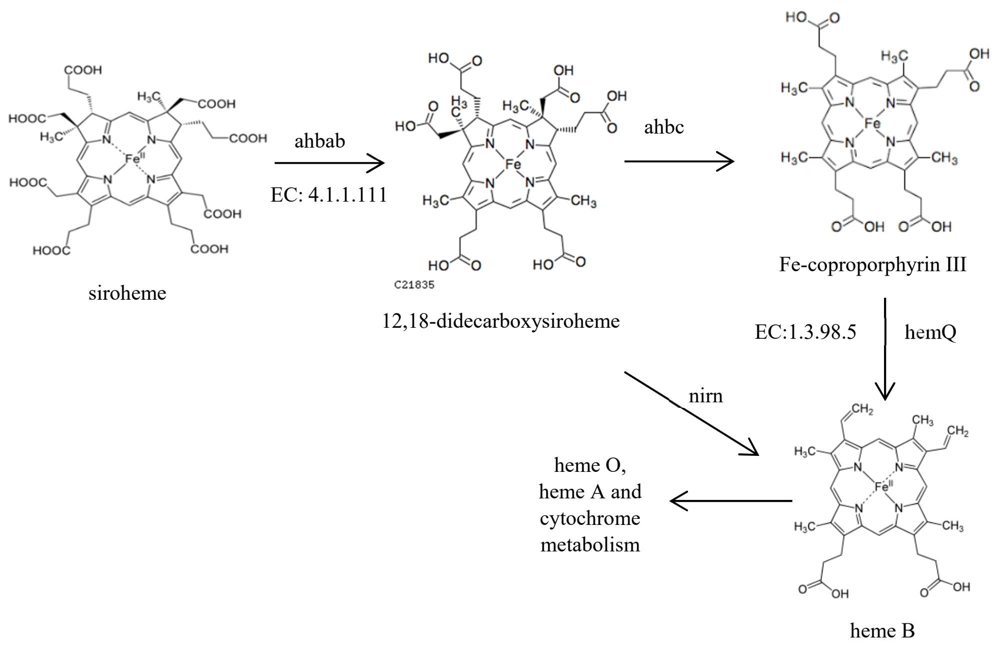

Figure S5. Biosynthetic pathway of siroheme-dependent heme

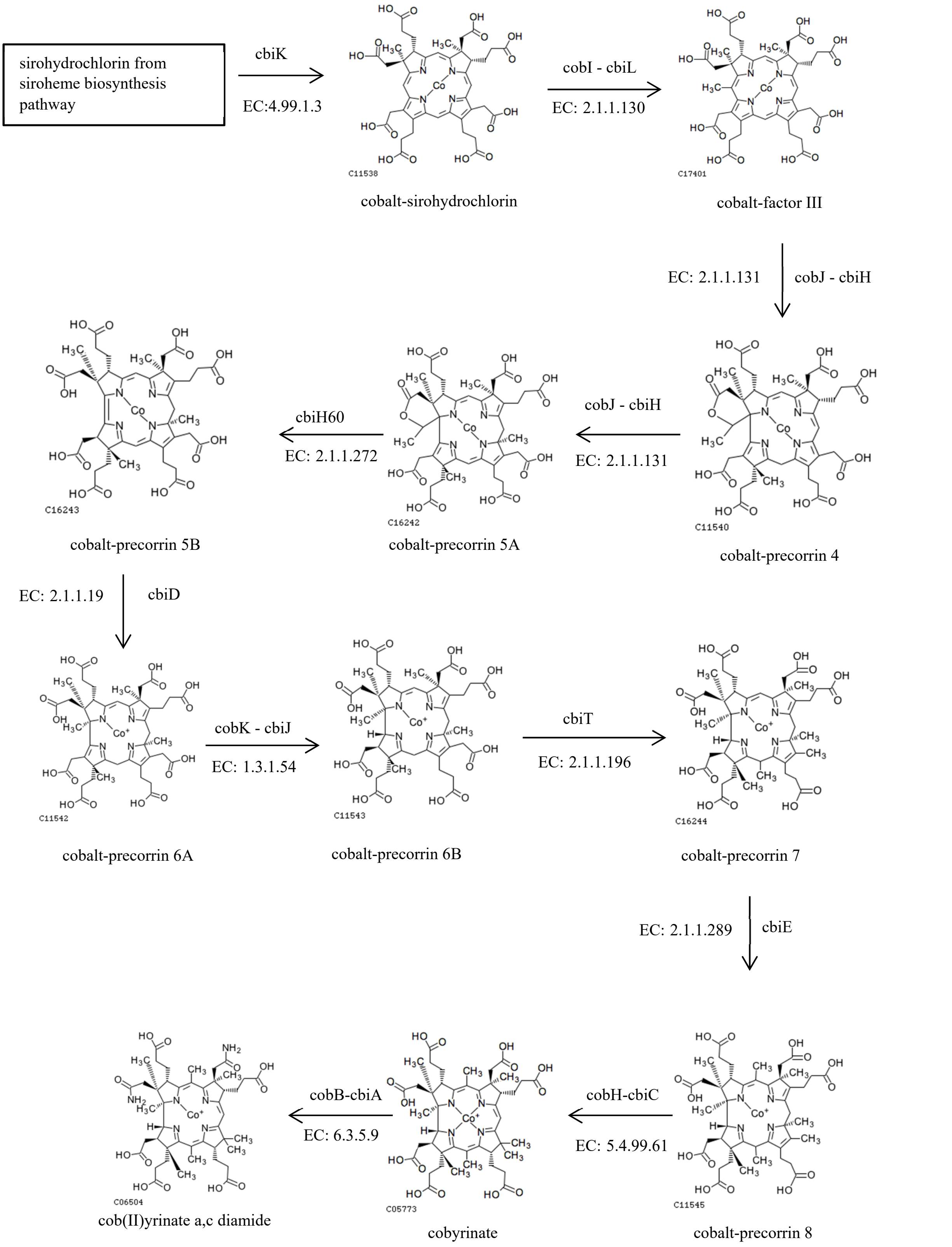

Figure S6. Biosynthetic pathway of anaerobic cob(II)yrinate a,c-diamide

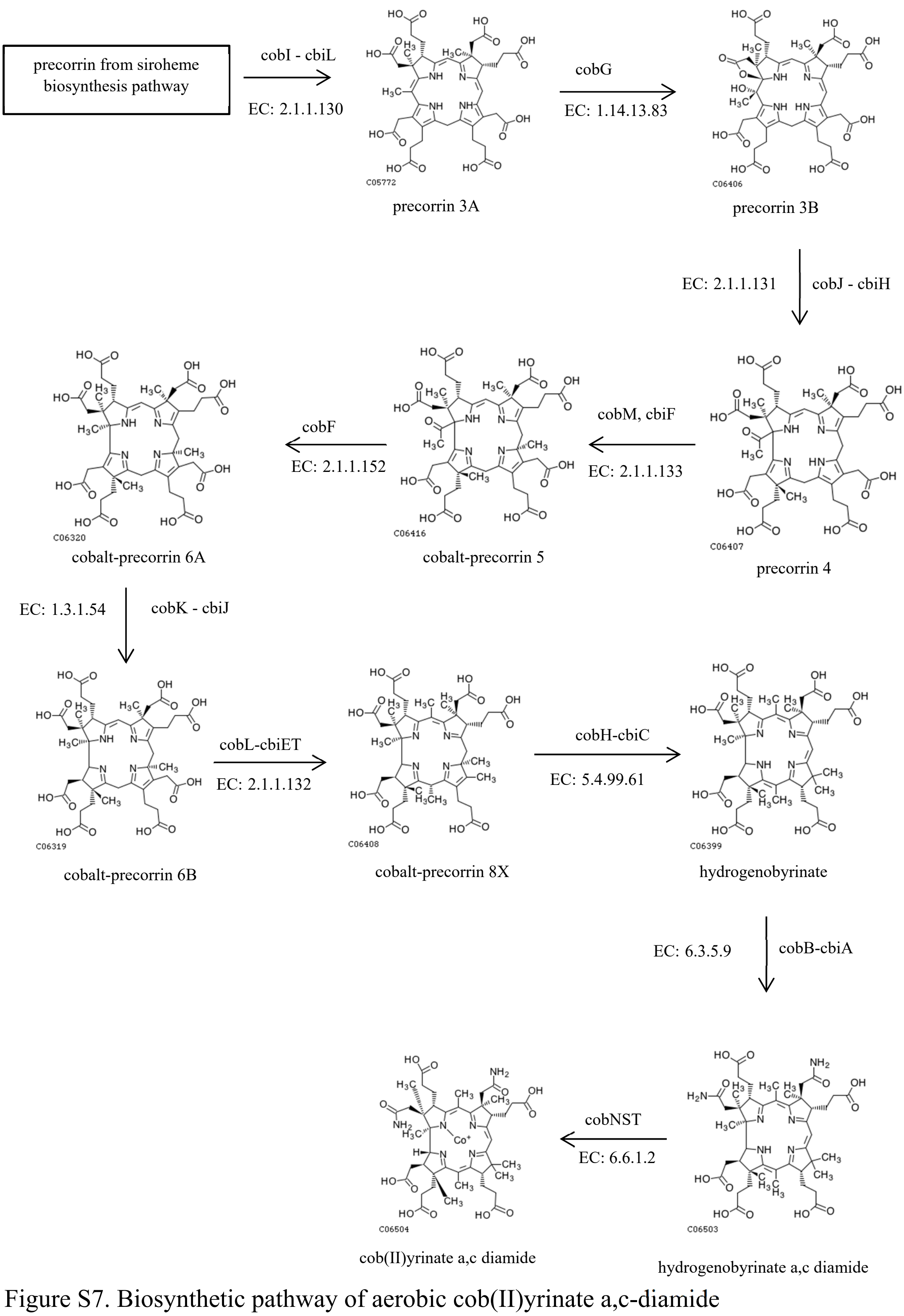

Figure S7. Biosynthetic pathway of aerobic cob(II)yrinate a,c-diamide

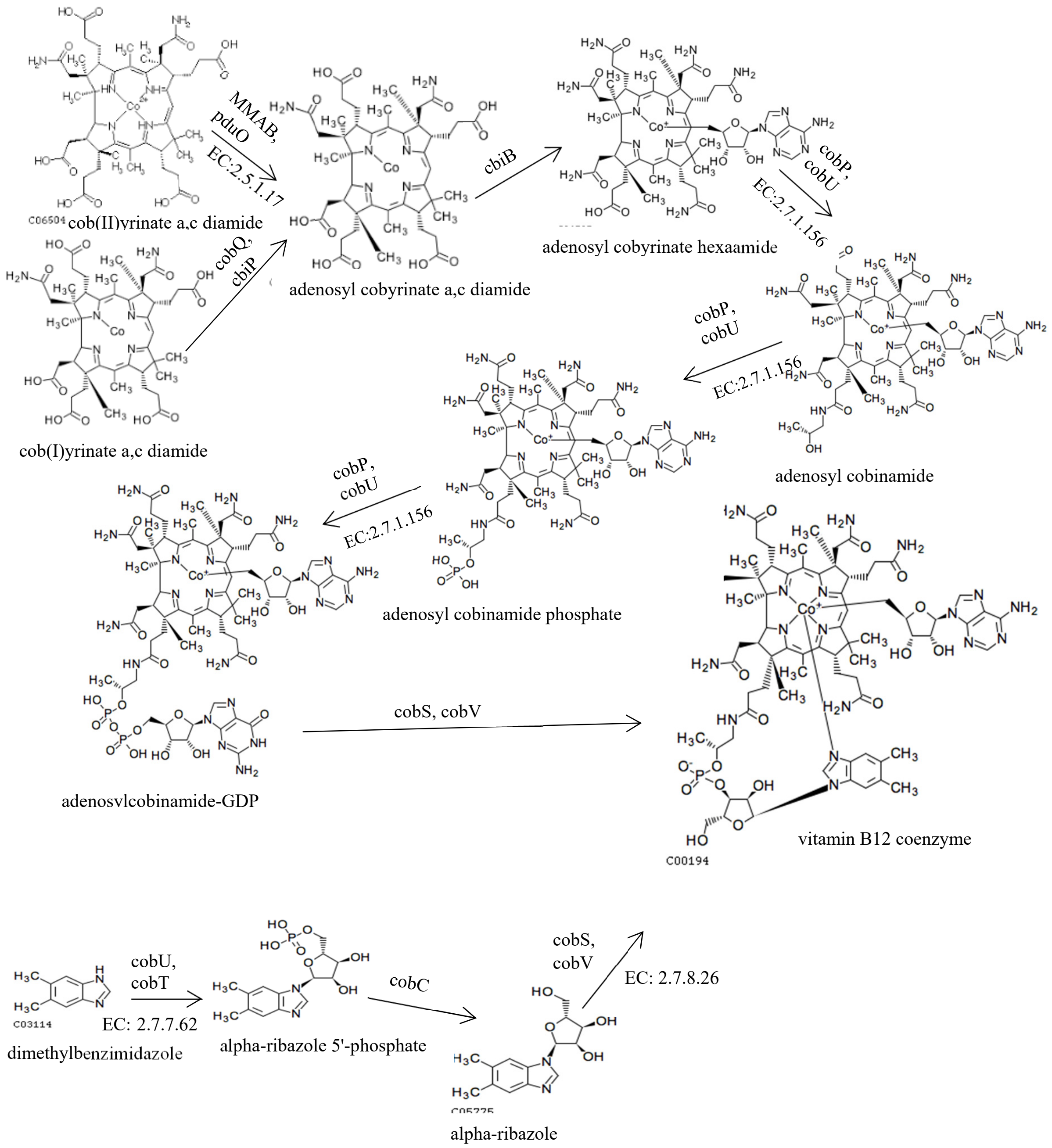

Figure S8. Biosynthetic pathway of cobalamin from cob(II)yrinate a,c-diamide
