# Supplementary material for: Biosynthesis of Tetrapyrrole Cofactors by Bacterial Community Inhabiting Porphyrine-Containing Shale Rock (Fore-Sudetic Monocline)
Source: Molecules. 2021 Nov 8;26(21):6746. doi: 10.3390/molecules26216746 (PMC8587615; doi:10.3390/molecules26216746)
Supplement: Supplementary file 1 [file molecules-26-06746-s001.zip › Supplementary Material Table S3. Proteins detected in the metaproteome of bacterial community involved in the biosynthetic pathways under study.pdf]

Proteins detected in the metaproteome of bacterial community involved in the biosynthetic pathways under study

Table S3. List of protein sequences detected in the metaproteome of bacterial community involved in biosynthetic pathways under study

Name of enzyme	Accession (NCBI)	Organism
**Uroporphyrinogen III biosynthesis pathway**		
porphobilinogen synthase	WP_002458905.1	*Staphylococcus* sp.
	WP_008011359.1	*Pseudomonas* sp. GM50
	WP_013972900.1	*Pseudomonas* sp.
	WP_003211635.1	*Pseudomonas* sp.
	WP_009048237.1	*Pseudomonas chlororaphis*
hydroxymethylbilane synthase	WP_006599024.1	*Pseudoramibacter alactolyticus*
	WP_011275555.1	*Staphylococcus* sp.
	WP_019341266.1	*Pseudomonas stutzeri*
	WP_003379385.1	*Pseudomonas syringae*
	WP_007473976.1	*Caminibacter mediatlanticus*
	WP_004408797.1	*Pseudomonas syringae*
	WP_014821749.1	*Pseudomonas stutzeri*
	gi|493647429	*Pseudoramibacter alactolyticus*
**Siroheme biosynthesis pathway**		
uroporphyrin-III C-methyltransferase	WP_173512851.1	*Ensifer psoraleae*
	WP_150296063.1	*Pseudomonas extremaustralis*
	WP_083357327.1	*Pseudomonas antarctica*
	WP_110622650.1	*Pseudomonas* sp. OV286
	WP_198862205.1	*Pseudomonas carnis*
	WP_080519948.1	*Pseudomonas tolaasii*
	WP_028011927.1	*Sinorhizobium meliloti*
	WP_014764895.1	*Sinorhizobium fredii*
uroporphyrin-III C-methyltransferase / precorrin-2 dehydrogenase / sirohydrochlorin ferrochelatase	WP_117436720.1	*Mycobacterium marinum*
	WP_198965845.1	*Mycobacterium shottsii*
	WP_179694435.1	*Pseudomonas moraviensis*
	WP_127798710.1	*Pseudomonas syringae*
	WP_085613263.1	unclassified Pseudomonas
	WP_192561590.1	*Pseudomonas* sp. IzPS32d
	WP_064597721.1	*Pseudomonas* sp. DR 5-09
	WP_108562679.1	*Pseudomonas fluorescens*
**Protoporphyrin-dependent heme biosynthesis pathway**		
uroporphyrinogen decarboxylase	WP_011275429	*Staphylococcus* sp.
	WP_002472704	*Staphylococcus pettenkoferi*
	NP_253721	*Pseudomonas aeruginosa* PAO1
	WP_026853903	*Geothrix fermentans*
coproporphyrinogen-III oxidase	NP_250237	*Pseudomonas aeruginosa* PAO1
oxygen-independent coproporphyrinogen III oxidase	gi|659868130	*Caulobacter* sp. UNC358MFTsu5.1
	WP_010561650.1	*Pseudoalteromonas spongiae*

	WP_008165597.1	*Pseudoalteromonas* BSi20652
	WP_008165597.2	*Pseudoalteromonas* sp. BSi20429
protoporphyrin/coproporphyrin ferrochelatase	HCQ13473.1	*Pseudomonas protegens*
	WP_011275430.1	*Pseudomonas protegens*
	gi|499594696	*Pseudomonas protegens*
	WP_011275430.1	*Pseudomonas protegens*
	gi|499594696	*Pseudomonas protegens*
**Coproporphyrin -dependent heme biosynthesis pathway**		
protoporphyrin/coproporphyrin ferrochelatase	HCQ13473.1	*Pseudomonas protegens*
	WP_011275430.1	*Pseudomonas protegens*
	gi|499594696	*Pseudomonas protegens*
	WP_011275430.1	*Pseudomonas protegens*
	gi|499594696	*Pseudomonas protegens*
**Siroheme-dependent heme biosynthesis pathway**		
Fe-coproporphyrin III synthase	WP_114516816.1	*Ensifer sesbaniae*
	WP_103784311.1	*Ensifer adhaerens*
	WP_198561225.1	*Pseudomonas* sp. OV286
**Anaerobic cob(II)yrinate a, c diamide biosynthesis pathway**		
cobyrinic acid a,c-diamide synthase	KAJ26206.1	*Pseudomonas aeruginosa* M10
	WP_193067791.1	unclassified *Halomonas*
cobalt-precorrin-5B (C1)-methyltransferase	WP_079682569.1	*Parabacteroides chartae*
**Aerobic cob(II)yrinate a, c diamide biosynthesis pathway**		
cobyrinic acid a,c-diamide synthase	KAJ26206.1	*Pseudomonas aeruginosa* M10
	WP_193067791.1	unclassified *Halomonas*
**Cobalamin biosynthesis pathway from cob(II)yrinate a,c diamide**		
cob(I)alamin adenosyltransferase	WP_171978626.1	*Pseudomonas aeruginosa*
	WP_121499914.1	*Pseudomonas aeruginosa*
adenosylcobinamide kinase / adenosylcobinamide-phosphate guanylyltransferase	WP_173516816.1	*Ensifer sesbaniae*
	WP_104664311.1	*Ensifer adhaerens*
	WP_077960925.1	*Ensifer adhaerens*
	WP_077503836.1	*Sinorhizobium* sp. A49
	KSV90196.1	*Sinorhizobium* sp. GL28
nicotinate-nucleotide--dimethylbenzimidazole phosphoribosyltransferase	WP_046617183.1	*Pseudomonas putida*
	WP_075044018.1	*Pseudomonas putida*
	WP_085706506.1	*Pseudomonas* sp. B8(2017)
**Transport of porphyrins**		
periplasmic protein TonB	WP_197862675.1	*Pseudomonas putida*
biopolymer transport protein ExbB	WP_164634537.1	*Rhodopseudomonas* sp. BR0G17
biopolymer transport protein ExbD	WP_072840788.1	*Marinomonas polaris* DSM 16579
